# Complexation of Plutonium and Other Actinides in Different Oxidation States with Gluconate at Low pH Values—A CE-ICP-MS Study

**DOI:** 10.1021/acs.inorgchem.5c04403

**Published:** 2026-02-10

**Authors:** Janik Lohmann, Felix Sprunk, Diana Velikotrav, Alexander Wiebe, Julia Zemke, Tobias Reich

**Affiliations:** Department of Chemistry-Nuclear Chemistry, 9182 Johannes Gutenberg-Universität Mainz, Mainz 55099, Germany

## Abstract

Using a coupling between capillary electrophoresis and ICP-MS (CE-ICP-MS), the gluconate (GLU) complexation of plutonium in the major oxidation states (III)–(VI) as well as Am­(III), Th­(IV), Np­(V), and U­(VI) was investigated at pH ≤ 4. CE-ICP-MS enabled the determination of the Pu oxidation state by comparing its electrophoretic mobility to that of a redox-analogous actinide (An). For the Am­(III)/Pu­(III) pair, the complex formation constants of three successive binary [An­(GLU)_*x*_]^3–*x*^ (*x* = 1–3) complexes could be determined. For Np­(V)/Pu­(V), the complex formation constants of the first binary [AnO_2_(GLU)]_(aq)_ complex were determined in accordance with previous literature for Np­(V), and those of the second [AnO_2_(GLU)_2_]^−^ complex were estimated. For U­(VI)/Pu­(VI), the constants of the [AnO_2_(GLU)]^+^, [AnO_2_(GLU_–H_)]_(aq)_, and [AnO_2_(GLU_–H_)­(GLU)]^−^ complexes were also determined in accordance with previous literature for U­(VI). Plutonium in the oxidation states (III), (V), and (VI) behaved very similarly to the redox analogues. This was not the case for Th­(IV)/Pu­(IV). Here, the first five binary [Th­(GLU)_*x*_]^4–*x*^ (*x* = 1–5) complexes were determined for Th­(IV), whereas mixed Pu–OH–GLU complexes were proposed for Pu­(IV). The comparison of the first complex formation constants of the binary An–GLU complexes suggests a different bonding motif between An^3+/4+^ and AnO_2_^+/2+^, with AnO_2_^+/2+^ forming the weaker complexes.

## Graphical abstract

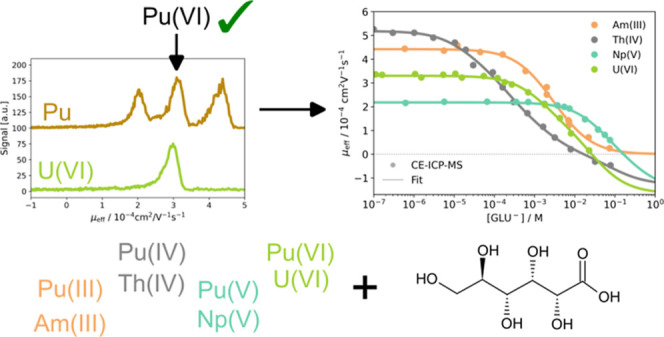

## 1 Introduction

Gluconic acid (Figure 1) is considered to be one of the most important organic ligands that could influence the mobility of radionuclides in the near field of a potential low or intermediate level nuclear waste repository.^1^

**Figure 1:**
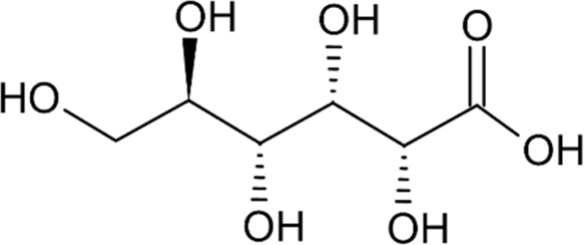
Molecular structure of d-gluconic acid.

Through its use as a potential cement additive, gluconate can be released during cement degradation. Several studies have already demonstrated the influence of gluconate on the retention of tri- and tetravalent actinides under conditions relevant to the nuclear waste repository.^2–6^ Furthermore, large quantities of sodium gluconate were introduced at the Hanford site as part of the Manhattan project.^7^

Plutonium is responsible for a large part of the radiotoxicity in spent fuels.^8^ Due to the complicated redox chemistry, studies with plutonium require a high degree of redox control.^9^ This is probably one of the reasons that there are only a few studies with Pu, especially at low pH values. In the literature, mainly redox-stable actinides have been investigated as analogues of Pu in different oxidation states.

Most studies regarding the influence of gluconate under repository-relevant conditions were conducted at alkaline or hyperalkaline pH values, which are expected during cement aging. Under the reductive conditions in the repository, actinides predominantly occur in the oxidation states +III and +IV. In literature, gluconate complexation has therefore mainly been investigated with Th­(IV)^3,5,10^ or U­(IV)^11^ as a representative of An­(IV) and with Am­(III),^10^ Cm­(III),^6^ or Eu­(III)^10^ as representatives of An­(III). For An­(VI), the complexation of U­(VI)^12,13^ with gluconate was investigated. To our knowledge, no studies on the complexation of An­(V) with GLU in an alkaline solution exist. The complexation of plutonium with GLU was only ever investigated for Pu­(IV).^5^ At high pH values, hydrolysis of An and the formation of mixed An–OH–GLU complexes occur.^5,6,14^ Although the second p*K*_a_ of gluconic acid is approximately 13 ± 1,^15^ metal-induced ligand deprotonation can occur in the metal complex, resulting in the abstraction of one or more of the alcohol protons at significantly lower pH values.^14,16,17^ All of these effects complicate the determination of thermodynamic data.

In order to investigate mainly the binary An–GLU complexation, experiments in this work were performed at pH ≤ 4. The investigations were carried out using a combination of capillary electrophoresis (CE) and ICP-MS. In our previous work, good agreement between CE-ICP-MS and TRLFS was achieved for the Eu­(III)–GLU complexation.^14^

Zhang et al. systematically investigated the complexation of Nd­(III),^18^ Th­(IV),^7^ Np­(V),^19^ and U­(VI)^17^ with gluconate under similar conditions to this work using a combination of potentiometry, spectrophotometry, nuclear magnetic resonance (NMR) spectroscopy, and extended X-ray absorption fine structure (EXAFS) studies. They described the formation of binary complexes with up to three gluconate ligands for Nd­(III),^18^ two for Np­(V),^19^ and one for U­(VI).^17^ For Th­(IV)^7^ and U­(VI),^17^ Zhang et al. proposed the formation of hydroxyl-deprotonated gluconate complexes even at low pH values.

In this work, the ability of CE-ICP-MS to measure multiple analytes simultaneously was used to confirm the desired Pu oxidation state by comparing the electrophoretic mobility with that of a redox-stable actinide. Thus, gluconate complexation was investigated for the pairs Am­(III)/Pu­(III) and Np­(V)/Pu­(V) at pH 4, U­(VI)/Pu­(VI) at pH 3, and Th­(IV)/Pu­(IV) between pH 1.3 and 2.7.

## 2 Experimental Section

### 2.1 Reagents

Caution! ^232^Th, ^237^Np, ^238^U, ^239^Pu, and ^241^Am are radioactive elements and require special precautions as well as radiation protection. Never evaporate conc. HClO_4_ solutions to complete dryness.

All chemicals used were of analytical grade or better, except for gluconic acid, 50% aq. soln. (Fisher Scientific GmbH, Schwerte, Germany). Milli-Q water was used throughout all experiments (18.2 MΩcm, Synergy Millipore water system, Millipore GmbH, Schwalbach, Germany).

The plutonium stock solutions were prepared by starting from a ^239^Pu­(IV) stock solution. This stock was prepared by electrolysis in 1 M HClO_4_. A detailed description of the process is given in Stietz et al.^20^ For the Pu­(IV) series, this stock was used as is.

Pu­(III) was prepared by mixing the Pu­(IV) stock with a U­(IV) stock at a molar ratio of 1:2. The plutonium was reduced to Pu­(III) based on the following redox reaction:^9^

1 $$U^{4+}+2{Pu}^{4+}+2H_{2}O⇌UO_{2}^{2+}+2Pu^{3+}+{4H}^{+}$$

The U­(IV) stock was produced by dissolving metallic uranium in 8 M HCl (VWR Chemicals, Radnor, Pennsylvania, USA).

Pu­(V) and Pu­(VI) were both prepared by evaporation of the Pu­(IV) stock, first in concentrated HNO_3_ and then several times in 6 M HClO_4_ until near dryness to oxidize the plutonium to Pu­(VI). To produce the Pu­(VI) stock, the residue was added to 1 M HClO_4_. To produce the Pu­(V) stock, the residue was taken up in 0.1 M NaClO_4_ at a circumneutral pH value. The oxidation states were confirmed by UV–vis spectroscopy (Tidas 100, J&M Analytik AG, Essingen, Germany). The concentration of the ^239^Pu stock solutions was determined by liquid scintillation counting (LSC, Hidex 300 SL, Hidex, Finland) and α-spectrometry (Si surface barrier detector, CR-SNA-450-100, AMETEK, USA). All ^239^Pu stock solutions had a concentration of about 2 × 10^–4^ M.

The ^241^Am­(III) stock solution was prepared by evaporating an in-house ^241^Am solution to dryness and redissolving it in 0.1 M HClO_4_. For the ^237^Np­(V) stock solution, an in-house ^237^Np solution was evaporated until near dryness and redissolved in 1 M HClO_4_ (VWR, Darmstadt, Germany). This process was repeated three times to yield a Np­(VI) solution. In the last step, ^237^Np was dissolved in 0.1 M HClO_4_ and NaNO_2_ was added to reduce Np­(VI) to Np­(V). The oxidation state was confirmed by UV–vis (UV–vis) spectroscopy. The concentrations of the ^241^Am and ^237^Np solutions were determined by γ-ray spectroscopy (^241^Am at 59.5 keV, ^237^Np at 86.5 keV) using a high-purity germanium detector (GMX-13280-S, ORTEC, Oak Ridge, Tennessee, USA) and the Canberra InSpector 2000DSP Portable Workstation (Model IN2K, Canberra Industries Inc., Meriden, Connecticut, USA). The concentrations of the stock solutions were [^241^Am] = 3 × 10^–5^ M and [^237^Np] = 2 × 10^–4^ M. For the ^232^Th­(IV) and ^238^U­(VI) stock solutions, ICP-MS standards (^232^Th: Accu Trace, Accu Standard, New Haven, CT, USA, ^238^U: SPEX Certiprep, Metuchen, Massachusetts, USA) of known concentrations were evaporated and redissolved in a 0.1 M HClO_4_ producing stock solution with a concentration of 2 × 10^–4^ M, respectively.

### 2.2 Sample Preparation

For each measurement, 10 mL of an appropriate background electrolyte (BGE) with the desired GLU concentration (NaGLU for An­(III), An­(V), and An­(VI); HGLU and NaGLU for An­(IV)), pH value, and ionic strength was prepared. The ionic strength was fixed at 0.1 M and adjusted by using NaClO_4_. The pH values were adjusted using HClO_4_ and carbonate-free NaOH. The pH values were measured with a pH meter and a microelectrode (inoLab pH 720, Xylem, Weilheim, Germany, equipped with an SI AnalyticsBlueLine 16 pH microelectrode, Mainz, Germany, 3 M NaCl). The device was calibrated with reference buffer solutions at pH 4.01, pH 6.87, and pH 9.18 (Certipur, Merck, Darmstadt, Germany).

Samples of the Am­(III)/Pu­(III) and Np­(V)/Pu­(V) systems were prepared at pH 4 under an Ar atmosphere in analogy to the preparation described in Zenker et al.^14^ The U­(VI)/Pu­(VI) samples at pH 4 did not produce satisfying signals; therefore, the samples were prepared at pH 3 under ambient air conditions. Due to the hydrolysis of An­(IV), the samples of the Th­(IV)/Pu­(IV) series were prepared mainly at pH 1.3 under ambient air conditions.

Aliquots of the BGEs were mixed with the corresponding An stock solutions either by previously evaporating an aliquot of the stock and redissolving it in the BGE (An­(III) and An­(VI)) or by direct addition of the stock to the BGE (An­(IV) and An­(V)). Either way, no significant change in the pH value was observed after the addition. The final actinide concentrations were about 3 × 10^–8^ M for Am­(III) and about 2 × 10^–7^ M for all other actinides. As an internal standard with constant electrophoretic mobility, 1 × 10^–6^ M Cs^+^ was added. For a direct comparison with Zenker et al.,^14^ 1 × 10^–6^ M Eu­(III) was added to the An­(IV) samples.

Samples were measured on the same day as prepared. Especially the An­(VI) samples were measured immediately after preparation, as the reduction of Pu­(VI) took place within minutes.

For the CE measurements, 1 μL of 2-bromopropane (Merck, Darmstadt, Germany) was added to 200 μL of each actinide sample as a neutral marker. Prior to each measurement, the capillary was flushed using a BGE of the same composition as the sample. Actinide samples were injected hydrodynamically at 100 mbar for 5 s.

### 2.3 CE-ICP-MS

All CE measurements were performed using an Agilent 7100 CE system (Agilent, Santa Clara, California, USA) hyphenated to an Agilent 7900 ICP-MS system (Agilent, Santa Clara, California, USA). The coupling was realized via a MiraMist CE Nebulizer (Burgener Research, Mississauga, Canada) and a Scott-type spray chamber (AHS Analysentechnik, Tübingen, Germany). A fused silica capillary (TSP0503753, Polymicro Technologies, Phoenix, Arizona, USA) with a 50 μm inner diameter and a 50 cm length was used. A voltage of +10 kV and a pressure of 90 mbar were applied to aid the electro-osmotic flow (EOF). The temperature was kept at 25.0 ± 0.1 °C using internal air cooling of the CE device as well as a custom-built enclosure for the hyphenation.

### 2.4 Determination of Complexation Constants by Capillary Electrophoresis

From the CE measurements, migration times of the actinides and the neutral marker for the EOF were determined. The effective electrophoretic mobility μ_eff_ can be calculated by eq 2 with the migration time of the actinide *t*_An_, the migration time of a neutral marker *t*_EOF_, indicating the EOF, the effective length *l* of the capillary, and the applied voltage *U*.

2 $$\mu_{eff}=\frac{l^{2}}{U}\left(\frac{1}{t_{An}}-\frac{1}{t_{EOF}}\right)$$

The determination of complex formation constants of the An–GLU complexes was performed analogously to the procedure described in Zenker et al.^14^ for the determination of the Ln­(III)–GLU complexes.

The equilibria of actinides An^*z*+^ (An­(III) and An­(IV)) and AnO_2_^*z*+^ (An­(VI) and An­(VI)) with gluconate shown in eqs 3 and 4 are assumed.

3 $${An}^{z+}+i{GLU}^{-}⇌An{\left(GLU\right)}_{i}^{z-i}$$

4 $$AnO_{2}^{z+}+i{GLU}^{-}⇌AnO_{2}{\left(GLU\right)}_{i}^{z-i}$$

Based on the equilibria in eqs 3 and 4, eq 5 was drawn up to describe μ_eff_ in relation to the free gluconate concentration [GLU^–^] as well as the individual mobilities of the species μ_*i*_.

5 $$\mu_{eff}=\frac{\mu_{0}+\sum_{i=1}^{N}\mu_{i}\beta_{i}{\left[{GLU}^{-}\right]}^{i}}{1+\sum_{i=1}^{N}\beta_{i}{\left[{GLU}^{-}\right]}^{i}}$$

The mobilities of the individual species μ_*i*_ were estimated based on the quotient *Q* of the ionic charge *z* and the electrophoretic mobility, described in Lohmann et al.^21^ Values for *Q* were determined by using the measured electrophoretic mobility of the free actinide cations at low gluconate concentrations. For the succeeding An–GLU complexes, μ_*i*_ was estimated based on the ionic charge of the complex. All values for *Q* and μ_i_ are summarized in Table S3, Supporting Information.

Gluconic acid forms a γ-lactone and a δ-lactone by esterification at low pH values.^15,22^ In previous literature, the free gluconate concentration has often been calculated only using the p*K*_a_ value. In their extensive work, Zhang et al. justified this approach using the kinetics of the lactonization and the fast preparation and measurement of their samples.^7,17–19^ In the present work, BGEs were prepared at least one day prior to the measurements. It can be assumed the lactone was already in equilibrium.^15^ To check for the influence of the lactonization, the Eu­(III)–GLU constants were determined with and without consideration of lactonization at pH 1.3 – 2.76 (the lowest pH values in this work) and compared to the data at pH 4 published in Zenker et al.^14^ The inclusion of the lactone in the thermodynamical model does not influence the results significantly, as shown in Figures S1 and S2 as well as Table S1, Supporting Information. Therefore, in accordance with the ThermoChimie database (V13a),^23^ [GLU^–^] was determined using only the p*K*_*a*_ value of 3.7 at *I* = 0.1 M (NaClO_4_) determined by Zubiaur et al.^22^

In the electropherograms of Pu, often, several peaks were observed, indicating the simultaneous presence of multiple oxidation states (Figure 2). To assign the correct peak to the corresponding Pu oxidation states, the redox stable actinides Am­(III), Th­(IV), Np­(V), and U­(VI) were added to the samples. To limit the number of analytes, only one oxidation state was investigated at a time, e.g., Pu­(VI) and U­(VI). Although data for several Pu oxidation states were collected in each series, only peaks in clear correlation to the added redox analogue were considered for the evaluation. All electropherograms are shown in Figures S7–S11, Supporting Information. The calculated mobilities based on eq 2 along with the experimental parameters are listed in Tables S4–S8, Supporting Information.

**Figure 2:**
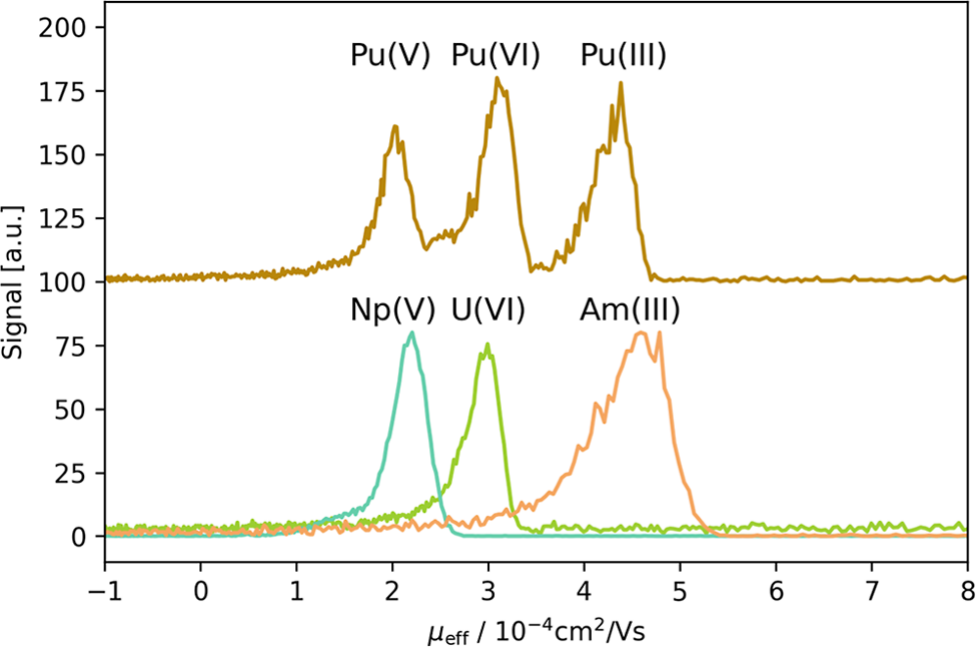
Electropherograms of Pu and U­(VI) (2 × 10^–4^ M NaGLU at pH 3), Am­(III) (1 × 10^–6^ M NaGLU at pH 3), and Np­(V) (1 × 10^–4^ M NaGLU at pH 4), superimposed.

By fitting eq 5 to the experimental data, the complex formation constants log β_*i*_ were obtained. All complex formation constants were extrapolated from 0.1 M to zero ionic strength using the Davies equation.^24^

## 3 Results and Discussion

### 3.1 Determination of Complexation Constants

#### 3.1.1 Am­(III)/Pu­(III) Gluconate

The measured electrophoretic mobilities of Am­(III) and Pu­(III) at pH 4 as a function of the free gluconate concentration [GLU^–^] are shown in Figure 3. Both actinides exhibit a reduction in electrophoretic mobility with increasing gluconate concentration caused by the formation of An­(III)–GLU complexes and, thus, a reduction in mean ionic charge. Under the experimental conditions, the electropherograms measured at low GLU concentrations were dominated by peaks of Pu­(III) with a similar electrophoretic mobility as of Am­(III) (Figure S7, Supporting Information). With an increase in GLU concentration, peaks with neutral or negative mobilities dominated, potentially corresponding to Pu­(IV)–OH–GLU species. Despite that, nearly all samples showed a peak of Pu­(III) with a mobility similar to that of Am­(III).

**Figure 3:**
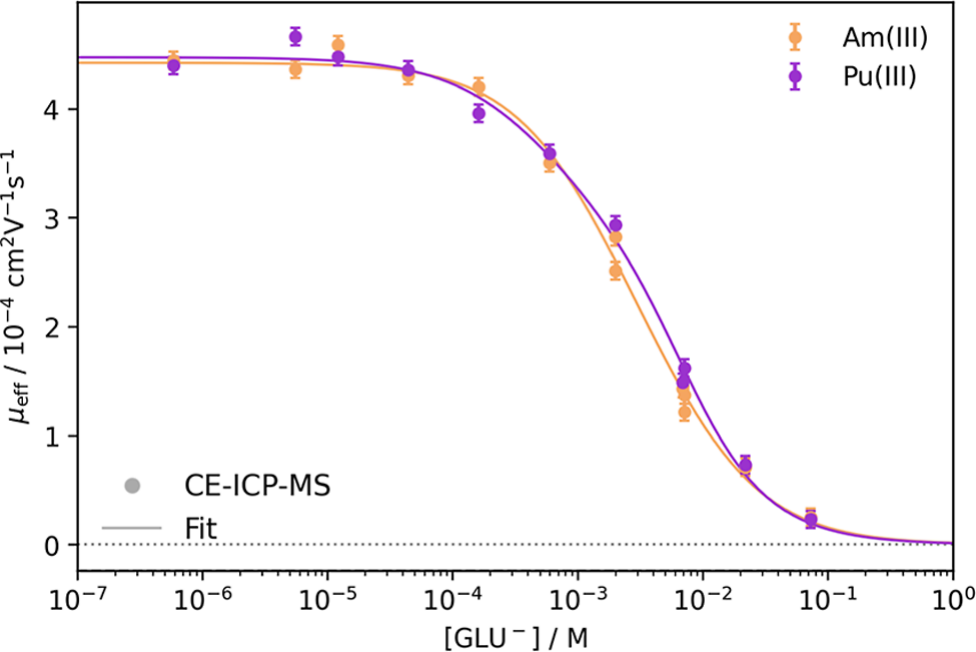
Plot of the measured electrophoretic mobilities μ_eff_ of ^241^Am­(III) and ^239^Pu­(III) against the free gluconate concentration [GLU^–^] at pH 4 and *I* = 0.1 M (NaClO_4_). Fits include the 1:1 through 1:3 An­(III)–GLU complexes using eq 5; *R* ^2^_Am(III)_ = 0.995 and *R* ^2^_Pu(III)_ = 0.996.

Using eq 5, the experimental data were fitted considering three successive An­(III)–GLU complexes. The complex formation constants at *I* = 0.1 M and extrapolated to zero ionic strength using the Davies equation^24^ are summarized in Table 1.

**Table 1:** Complex Formation Constants Log β^*I*=0.1M^ of the An­(III)–GLU Complexes of Am­(III) and Pu­(III) Obtained from the Fitting Procedure at *I* = 0.1 M (NaClO_4_) and ϑ = 25 °C, as Well as log β^0^ Extrapolated to Zero Ionic Strength Using the Davies Equation^24^

	${\log\beta }^{I=0.1M}$		${\log\beta }^{0}$	
	Am(III)	Pu(III)	Am(III)	Pu(III)
[An(GLU)]^2+^	3.09 ± 0.14	3.28 ± 0.12	3.73 ± 0.14	3.92 ± 0.12
[An(GLU)_2_]^+^	5.62 ± 0.09	5.49 ± 0.14	6.69 ± 0.09	6.56 ± 0.14
[An(GLU)_3_]_(aq)_	7.57 ± 0.11	7.52 ± 0.11	8.85 ± 0.11	8.80 ± 0.11

Am­(III) and Pu­(III) exhibit similar complex formation constants within the margin of error. Based on the similar ionic radii of Pu­(III), Am­(III), and Eu­(III),^25^ the latter can be used as a nonradioactive analog to An­(III). The log β^0^ values of the three successive An­(III)–GLU complexes are in good agreement to the values for Eu­(III) determined using CE-ICP-MS by the authors in Zenker et al.^14^ under similar experimental conditions. The log β_*i*_^0^ values of the first three Eu­(III)–GLU complexes were 3.98 ± 0.04, 7.04 ± 0.05, and 8.91 ± 0.07, respectively. In Zenker et al.,^14^ a fourth negatively charged Ln­(III)–GLU complex was proposed for Eu­(III). For the trivalent actinides in the present work, it was not possible to determine such a complex during the fitting process as the mobility curve approaches zero at higher [GLU^–^]. The speciation diagrams corresponding to the proposed complexes are shown in Figure S13, Supporting Information.

#### 3.1.2 Np­(V)/Pu­(V) Gluconate

Under the experimental conditions, Np­(V) and Pu­(V) exhibited nearly identical behavior. No change in the oxidation state was observed for Pu­(V) in this set of experiments (Figure S9, Supporting Information). The measured electrophoretic mobilities of Np­(V) and Pu­(V) at pH 4 as a function of free gluconate concentration [GLU^–^] are shown in Figure 4. As seen for An­(III), both pentavalent actinides show a decrease in electrophoretic mobility with an increase in gluconate concentration.

**Figure 4:**
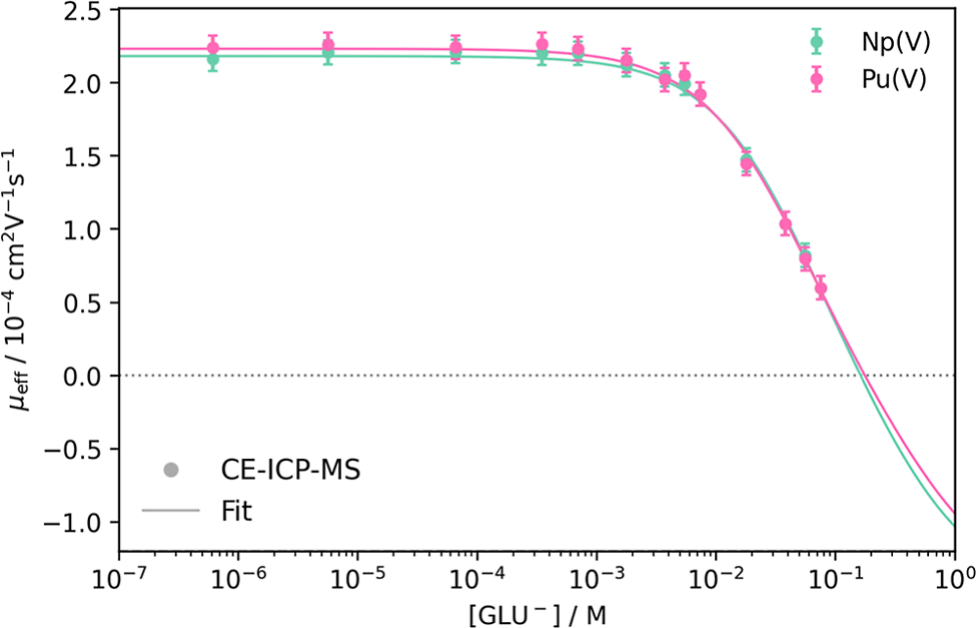
Plot of the measured electrophoretic mobilities μ_eff_ of ^237^Np­(V) and ^239^Pu­(V) against the free gluconate concentration [GLU^–^] at pH 4 and *I* = 0.1 M (NaClO_4_). Fits include the 1:1 and 1:2 An­(V)–GLU complexes using eq 5; *R* ^2^_Np(V)_ = 0.996 and *R* ^2^_Pu(V)_ = 0.996.

The electrophoretic mobility does not drop below zero, indicating only the presence of predominantly positive complexes under the experimental conditions. Therefore, only the ${\left[AnO_{2}\left(GLU\right)\right]}_{\left(aq\right)}$ complex was observed experimentally. Zhang et al.^19^ also observed the negatively charged ${\left[NpO_{2}{\left(GLU\right)}_{2}\right]}^{-}$ complex. As demonstrated in Zenker et al.,^14^ the complex formation constant of the subsequent ${\left[AnO_{2}{\left(GLU\right)}_{2}\right]}^{-}$ complex can be estimated by adding it to the fitting model and assuming a negative mobility. The results are summarized in Table 2.

**Table 2:** Complex Formation Constants log β^*I*=0.1M^ of the An­(V)–GLU Complexes of Np­(V) and Pu­(V) Obtained from the Fitting Procedure at *I* = 0.1 M (NaClO_4_) and ϑ = 25 °C, as Well as log β^0^ Extrapolated to Zero Ionic Strength Using the Davies Equation^24^

	log β^*I*=0.1M^		log β^0^	
	Np(V)	Pu(V)	Np(V)	Pu(V)
${\left[AnO_{2}\left(GLU\right)\right]}_{\left(aq\right)}$	1.34 ± 0.03	1.39 ± 0.03	1.55 ± 0.03	1.60 ± 0.03
${\left[AnO_{2}{\left(GLU\right)}_{2}\right]}^{-}$	1.74 ± 0.10	1.68 ± 0.12	1.95 ± 0.10	1.89 ± 0.12

Within the margin of error, the complex formation constants for Np­(V) and Pu­(V) are identical, as expected from their similar chemical behavior. The complex formation constant of the ${\left[NpO_{2}\left(GLU\right)\right]}_{\left(aq\right)}$ complex agrees within the margin of error with the value determined by Zhang et al.^19^ at *I* = 1 M (NaClO_4_) of 1.48 ± 0.03. This value was extrapolated to zero ionic strength (log β^0^ = 1.68 ± 0.10) using the Specific Ion Interaction Theory (SIT) approach in the latest Version (13a) of the ThermoChimie database^23^ (ion interaction coefficients ε­(*j*,*k*) are listed in Table S10). Assuming a negative mobility value (Table S3, Supporting Information) for the ${\left[NpO_{2}{\left(GLU\right)}_{2}\right]}^{-}$ complex, a weaker complexation as proposed by Zhang et al.^19^ was observed. They proposed a value of 2.14 ± 0.09 at *I* = 1 M (NaClO_4_) and 2.39 ± 0.10 extrapolated to zero ionic strength using SIT by the ThermoChimie database^23^ (ε­(*j*,*k*) in Table S10). The speciation diagrams using the data obtained in the present work are shown in Figure S14, Supporting Information.

#### 3.1.3 U­(VI)/Pu­(VI) Gluconate

At pH 4, no An­(VI) peaks were detected due to possible sorption effects on the capillary. Therefore, the experiments were conducted at pH 3.

Pu­(VI) and U­(VI) also exhibited a decrease in electrophoretic mobility with increasing free gluconate concentration, as shown in Figure 5. The determination of the Pu­(VI) mobility proved to be difficult, as it was rapidly reduced to Pu­(V), Pu­(IV), and Pu­(III) (Figure S17, Supporting Information). Samples had to be measured within seconds after the addition of the BGE to produce satisfying Pu­(VI) signals (Figure S11, Supporting Information).

**Figure 5:**
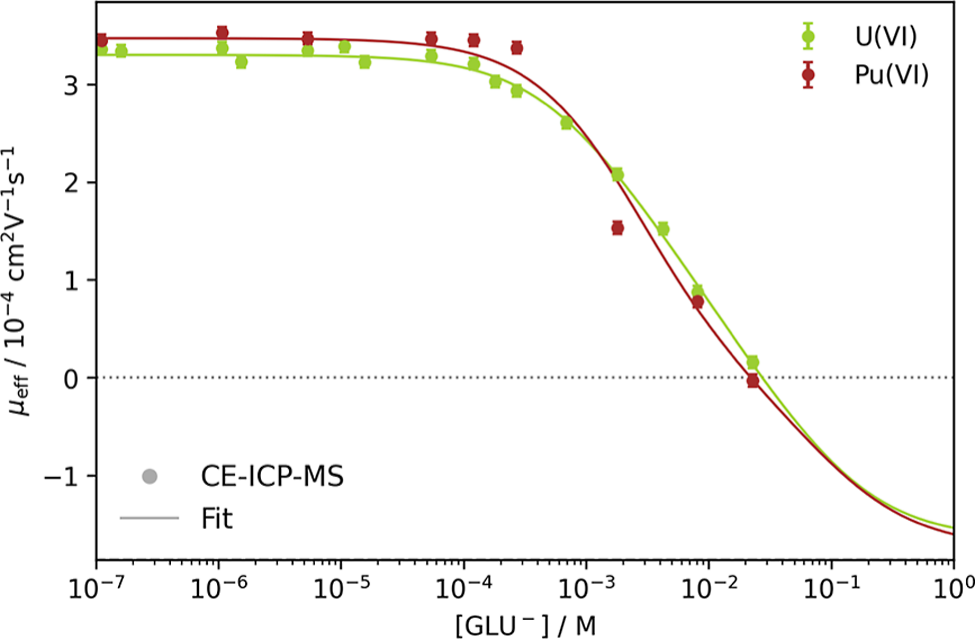
Plot of the measured electrophoretic mobilities μ_eff_ of ^238^U­(VI) and ^239^Pu­(VI) against the free gluconate concentration [GLU^–^] at pH 3 and *I* = 0.1 M (NaClO_4_). Fits include the AnO_2_(GLU)^+^, AnO_2_(GLU_–H_), and AnO_2_(GLU_–H_)­(GLU)^−^ complexes using eq 9; *R* ^2^_U(VI)_ = 0.996 and *R* ^2^_Pu(VI)_ = 0.982.

Applying the same procedure as for An­(III) and An­(V), the experimental data could be fitted assuming the formation of three ${\left[AnO_{2}{\left(GLU\right)}_{i}\right]}^{2-i}$ complexes (*i* = 1–3). The corresponding fit (Figure S4, Supporting Information) and complex formation constants (Table S2, Supporting Information) are shown in the Supporting Information.

Zhang et al.^17^ investigated the U­(VI) system in a similar pH range (2.5–4.2) at *I* = 1 M (NaClO_4_) using potentiometric, calorimetric, NMR, and EXAFS studies. Contrary to the formation of only the binary An­(VI)–GLU complex, they described the formation of the following three complexes:

6 $$UO_{2}^{2+}+{GLU}^{-}⇌{\left[UO_{2}\left(GLU\right)\right]}^{+}$$

7 $$UO_{2}^{2+}+{GLU}^{-}⇌\left[UO_{2}\left(GLU_{-H}\right)\right]+H^{+}$$

8 $$UO_{2}^{2+}+{2GLU}^{-}⇌{\left[UO_{2}\left(GLU\right)\left(GLU_{-H}\right)\right]}^{-}+H^{+}$$

The origin of the proton in eqs 7 and 8 is not fully understood yet, while the low pH values, where U­(VI) hydrolysis is not expected, strongly suggest metal-induced ligand deprotonation. The formation of a strong [UO_2_(GLU_–H_)] complex via a five-membered ring would reduce the second p*K*_a2_ of gluconate from around 13 to below 3. This effect has also been proposed for, e.g., Ca­(II)^16^ and Eu­(III).^14^ Independent of the origin of the proton, it needs to be included in the fit function:

9 $$\mu_{eff}=\frac{\mu_{0}+\sum \mu_{i,j}\beta_{i,j}{\left[{GLU}^{-}\right]}^{i}{\left[H^{+}\right]}^{-j}}{1+\sum \beta_{i,j}{\left[{GLU}^{-}\right]}^{i}{\left[H^{+}\right]}^{-j}}$$

The pH value of the measurements was 3.1 ± 0.1, and the fit is shown in Figure 5. Both assumptions (Figures 5 and S4) fit the data equally well. Thus, based on this experiment alone, it is impossible to confirm one of the models. Therefore, another experiment was performed for U­(VI) at a fixed free gluconate concentration at a varied pH value between 2 and 3.6 (Figure S5, Supporting Information). Since the equilibrium in eq 4 is not dependent on pH, no change in electrophoretic mobility is expected. Since a reduction in electrophoretic mobility was observed with increasing pH, indicating the formation of the [UO_2_(GLU_–H_)] and ${\left[UO_{2}\left(GLU_{-H}\right)\left(GLU\right)\right]}^{-}$ complexes, the equilibria proposed by Zhang et al.^17^ were confirmed in the present work. The calculated complex formation constants are summarized in Table 3. Since no literature is available for Pu­(VI), an analogous chemical behavior to that of U­(VI) was assumed.

**Table 3:** Complex Formation Constants log β^*I*=0.1M^ of the An­(VI)–GLU Complexes Obtained from the Fitting Procedure at *I* = 0.1 M (NaClO_4_) and ϑ = 25 °C, as Well as log β^0^ Extrapolated to Zero Ionic Strength Using the Davies Equation^24^

	log β^*I*=0.1M^		log β^0^	
	U(VI)	Pu(VI)	U(VI)	Pu(VI)
${\left[AnO_{2}\left(GLU\right)\right]}^{+}$	2.64 ± 0.24	2.50 ± 0.39	3.07 ± 0.24	2.93 ± 0.39
${\left[AnO_{2}\left(GLU_{-H}\right)\right]}_{\left(aq\right)}$	–0.83 ± 0.21	–0.49 ± 0.20	–0.40 ± 0.21	–0.06 ± 0.20
${\left[AnO_{2}\left(GLU_{-H}\right)\left(GLU\right)\right]}^{-}$	1.18 ± 0.25	1.17 ± 0.40	1.61 ± 0.25	1.60 ± 0.40

Within the margin of error, the complex formation constants for U­(VI) and Pu­(VI) are similar. The lack of reliable data points for Pu­(VI), due to the high tendency of reduction under the experiment parameters, results in higher uncertainty for the formation constants. The speciation diagrams corresponding to the proposed complexes are shown in Figure S15, Supporting Information.

The complex formation constant of the ${\left[UO_{2}\left(GLU\right)\right]}^{+}$ complex agrees within the margin of error with the value determined by Zhang et al.^17^ at *I* = 1 M (NaClO_4_) of 2.2 ± 0.3. This value was extrapolated to zero ionic strength (log β^0^ = 2.59 ± 0.30) using SIT in the latest Version (13a) of the ThermoChimie database (ε­(*j*,*k*) in Table S10). For the ${\left[UO_{2}\left(GLU_{-H}\right)\right]}_{\left(aq\right)}$ complex, Zhang et al. determined a formation constant of −0.38 ± 0.05 at *I* = 1 M (NaClO_4_). Based on this value, the ThermoChimie database selected a log β^0^ value of 0.20 ± 0.20. The value determined in the present work lies in the same order of magnitude, while the complexation was observed to be weaker compared to Zhang et al.^17^ For the ${\left[UO_{2}\left(GLU_{-H}\right)\left(GLU\right)\right]}^{-}$ complex, Zhang et al.^17^ determined a value of 1.3 ± 0.2 at *I* = 1 M (NaClO_4_). This value is not listed in the latest Version (13a) of the ThermoChimie database.^23^ Apart from the different ionic strengths, the value determined in the present work is close to that of Zhang et al.^17^

#### 3.1.4 Th­(IV)/Pu­(IV) Gluconate

To avoid the influence of Th­(IV) hydrolysis, the experiments were conducted between pH 1.3 and 2.76. In this pH range, Th­(IV) exists predominantly as Th^4+^ in the absence of any ligands. To obtain GLU^–^ concentrations of up to 0.1 M at these low pH values, without increasing the ionic strength, up to 0.75 M gluconic acid was used in the experiments. At gluconic acid concentrations >0.1 M, an influence of the viscosity of the samples on electrophoretic mobility was observed and corrected for, as described in the Figure S2 caption, Supporting Information. The measured electrophoretic mobilities as well as the corrected values for Th­(IV) and Pu­(IV) as a function of free gluconate concentration [GLU^–^] are shown in Figure 6. At low [GLU^–^], Pu­(IV) was partially reduced to Pu­(III) (Figure S8, Supporting Information).

**Figure 6:**
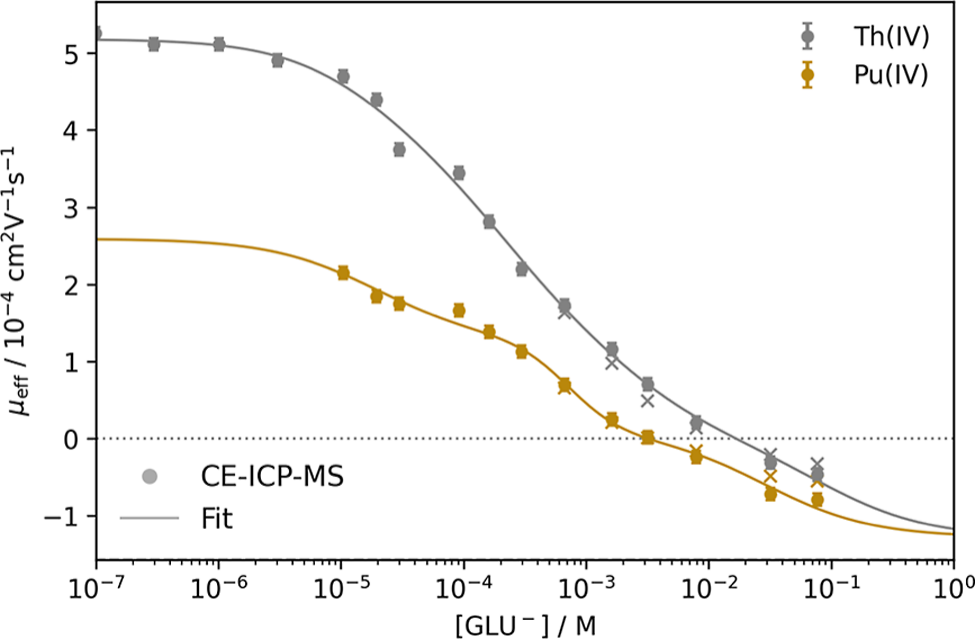
Plot of the measured electrophoretic mobilities μ_eff_ of ^232^Th­(IV) and ^239^Pu­(IV) against the free gluconate concentration [GLU^–^] at pH 1.3–2.76 and *I* = 0.1 M (NaClO_4_). The uncorrected values are marked by x. The fit of the Th data includes the 1:1 through 1:5 An­(IV)–GLU complexes using eq 5. The Pu­(IV) data were fitted using eq 10.

As can be seen in Figure 6, the chemical analogy between Pu and the redox analogue actinide, which was observed for An­(III), An­(V), and An­(VI), does not apply to Th­(IV) and Pu­(IV). Since Th is the furthest away from Pu in the periodic table compared to the other actinides studied, this is not surprising. For this reason, Th­(IV) and Pu­(IV) were treated separately.

For Th­(IV), Zhang et al.^7^ performed pD titrations and NMR spectroscopy in the pD range of 1.92–4.60 at [d-GLU^–^] ranging from 3 × 10^–4^ M to 4 × 10^–2^ M and *I* = 1 M (NaClO_4_). They proposed the formation of the ${\left[Th\left(GLU_{-D}\right)\right]}^{2+}$ and ${\left[Th\left(GLU_{-2D}\right)\right]}^{+}$ complexes. It is noted that due to the different ionic strength in this work (0.1 M) and in Zhang et al.^7^ (1 M), complexation cannot be compared one to one. To get a general idea, using their values accounted for the deuteration effect of log β_101(−1)_ = 0.54 and log β_101(−2)_ = −2.3,^7^ the gluconate complexation of Th­(IV) at pH 1.3 (as is the case in the present work) only occurs at [GLU^–^] > 5 × 10^–4^ M. As can be seen in Figure 6, a significant reduction in electrophoretic mobility was observed at [GLU^–^] > 1 × 10^–6^ M. At [GLU^–^] > 1 × 10^–2^ M, negative mobility values were observed, indicating the formation of negatively charged Th–GLU complexes. As most data points were determined at a constant pH of 1.3 and a significant reduction in mean charge from +4 to zero was observed, it is assumed that under the experimental conditions, no metal-induced ligand deprotonation occurs. The data was therefore fitted assuming the formation of five binary ${\left[Th{\left(GLU\right)}_{x}\right]}^{4-x}$ complexes (*x* = 1–5). The calculated complex formation constants are summarized in Table 4. It is noted that the data points corresponding to the ${\left[Th{\left(GLU\right)}_{5}\right]}^{-}$ complex were measured at increasing pH up to 2.76 and could have therefore been influenced by the potential onset of metal-induced ligand deprotonation.

**Table 4:** Complex Formation Constants log β^*I*=0.1M^ of the Th­(IV)–GLU Complexes Obtained from the Fitting Procedure at *I* = 0.1 M (NaClO_4_) and ϑ = 25 °C, as Well as log β^0^ Extrapolated to Zero Ionic Strength Using the Davies Equation^24^ ^,^ ^a^

	GLU		AcO^26^
	log β^*I*=0.1M^	log β^0^	log β^0^
[Th(L)]^3+^	4.81 ± 0.23	5.67 ± 0.23	5.06 ± 0.11
${\left[Th{\left(L\right)}_{2}\right]}^{2+}$	8.77 ± 0.24	10.27 ± 0.24	8.98 ± 0.20
${\left[Th{\left(L\right)}_{3}\right]}^{+}$	12.14 ± 0.25	14.07 ± 0.25	11.80 ± 0.50
${\left[Th{\left(L\right)}_{4}\right]}_{\left(aq\right)}$	14.68 ± 0.25	16.82 ± 0.25	13.90 ± 2.00
${\left[Th{\left(L\right)}_{5}\right]}^{-}$	15.72 ± 0.33	17.86 ± 0.33	-

a For comparison, log β^0^ values for the Th­(IV)–acetate complexes $\left({\left[Th{\left(AcO\right)}_{i}\right]}^{4-i}\right)$ determined by the authors are listed.^26^

The trend in log β^0^ of the Th–GLU complexes follows the trend in log β^0^ of the Th–AcO complexes determined in Lohmann et al.^26^ (Table 4). The complexation with gluconate is stronger by log β_GLU_^0^ = log β_AcO_^0^ + (0.69 ± 0.07) × *x* for ${\left[Th{\left(GLU\right)}_{x}\right]}^{4-x}$ (*x* = 1–4). The stronger complexation supports the multidentate bonding motif expected for Th–GLU complexes.^7^

In the pH range investigated, Pu­(IV) is predominantly present as the ${\left[Pu{\left(OH\right)}_{2}\right]}^{2+}$ species with 91% predominance at pH 1.70 to 69% at pH 2.76. Assuming the bonding of a maximum of five GLU^–^ ligands and neglecting metal-induced ligand deprotonation, 12 different Pu–OH–GLU complexes are plausible. As it is not feasible to include that many complexes in the fitting model, five complexes were selected based on the following assumptions (Figure S6, Supporting Information):

The number of OH^–^ ligands is estimated by the difference in the electrophoretic mobility between Th­(IV) and Pu­(IV) for a given [GLU^–^]. At low [GLU^–^], the difference in mobility is about 2 × 10^–4^ cm^2^ V^–1^ s^–1^, corresponding to a charge difference of about 2 between Th^4+^ and ${\left[Pu{\left(OH\right)}_{2}\right]}^{2+}$ . At high [GLU^–^], the difference is nearly zero, indicating the formation of similar complexes (Figure 6).

The number of GLU^–^ ligands is estimated based on the Th­(IV)–GLU complexation for a given [GLU^–^].

The assumed equilibria (log *K*, Table 5) all originate from the ${\left[Pu{\left(OH\right)}_{2}\right]}^{2+}$ complex. The fitting function was adapted as follows:

10 $$\mu_{eff}=\frac{\mu_{0}+\sum \mu_{i,-j}K_{i,-j}{\left[{GLU}^{-}\right]}^{i}{\left[H^{+}\right]}^{j}}{1+\sum K_{i,-j}{\left[{GLU}^{-}\right]}^{i}{\left[H^{+}\right]}^{j}}$$

**Table 5:** Estimated Complex Formation Constants log *K* ^*I*=0.1M^ of the Pu­(IV)–OH–GLU Complexes Obtained from the Fitting Procedure at *I* = 0.1 M (NaClO_4_) and ϑ = 25 °C

reaction	log *K* ^*I*=0.1M^
${\left[Pu{\left(OH\right)}_{2}\right]}^{2+}+{GLU}^{-}⇌{\left[Pu{\left(OH\right)}_{2}\left(GLU\right)\right]}^{+}$	4.71 ± 0.08
[Pu(OH)_2_]^2+^ + 2GLU^–^ + H^+^ ⇌ [Pu(OH)(GLU)_2_]^+^ + H_2_O	8.83 ± 1.40
${\left[Pu{\left(OH\right)}_{2}\right]}^{2+}+{3GLU}^{-}+H^{+}⇌{\left[Pu\left(OH\right){\left(GLU\right)}_{3}\right]}_{\left(aq\right)}+H_{2}O$	12.32 ± 0.23
${\left[Pu{\left(OH\right)}_{2}\right]}^{2+}+{4GLU}^{-}+{2H}^{+}⇌{\left[Pu{\left(GLU\right)}_{4}\right]}_{\left(aq\right)}+{2H}_{2}O$	16.27 ± 1.21
${\left[Pu{\left(OH\right)}_{2}\right]}^{2+}+{5GLU}^{-}+{2H}^{+}⇌{\left[Pu{\left(GLU\right)}_{5}\right]}^{-}+{2H}_{2}O$	19.59 ± 3.00

The results are summarized in Table 5. It is noted that the data points corresponding to the ${\left[Pu{\left(GLU\right)}_{5}\right]}^{-}$ complex were measured at increasing pH between pH 1.7 and 2.76. The change in pH increases the corresponding log *K* value by +2. Therefore, the overall uncertainty of the value was increased to ±3.00.

To obtain the log*β of the Pu­(IV)–OH–GLU complexes, the log *K* values in Table 5 were added to the log*β of ${\left[Pu{\left(OH\right)}_{2}\right]}^{2+}$ extrapolated to *I* = 0.1 M (−0.47 ± 0.30)^27^ using the Davies equation.^24^ The calculated complex formation constants are summarized in Table 6.

**Table 6:** Estimated Complex Formation Constants log*β^*I*=0.1M^ of the Pu­(IV)–OH–GLU Complexes at *I* = 0.1 M (NaClO_4_) and ϑ = 25 °C, as Well as log*β^0^ Extrapolated to Zero Ionic Strength Using the Davies Equation^24^

reaction	log*β^*I*=0.1M^	log*β^0^
${Pu}^{4+}+{2H}_{2}O+{GLU}^{-}⇌{\left[Pu{\left(OH\right)}_{2}\left(GLU\right)\right]}^{+}+{2H}^{+}$	4.24 ± 0.31	5.74 ± 0.31
${Pu}^{4+}+H_{2}O+{2GLU}^{-}⇌{\left[Pu\left(OH\right){\left(GLU\right)}_{2}\right]}^{+}+H^{+}$	8.36 ± 1.43	10.07 ± 1.43
${Pu}^{4+}+H_{2}O+{3GLU}^{-}⇌{\left[Pu\left(OH\right){\left(GLU\right)}_{3}\right]}_{\left(aq\right)}+H^{+}$	11.85 ± 0.38	13.78 ± 0.38
${Pu}^{4+}+{4GLU}^{-}⇌{\left[Pu{\left(GLU\right)}_{4}\right]}_{\left(aq\right)}$	15.80 ± 1.25	17.94 ± 1.25
${Pu}^{4+}+{5GLU}^{-}⇌{\left[Pu{\left(GLU\right)}_{5}\right]}^{-}$	19.12 ± 3.00	21.26 ± 3.00

Compared to the 1:4 and 1:5 Th­(IV)–GLU complexes, the estimated values for Pu­(IV) seem plausible. The complexation is stronger for Pu­(IV) as expected based on the higher charge density of the Pu^4+^ cation. The speciation diagrams corresponding to the proposed complexes are shown in Figure S16, Supporting Information.

It must be noted that the proposed Pu­(IV) complexes best match the experimental findings. To validate these assumptions, further experimental methods and DFT calculations are necessary to determine the complexation behavior and preferred stoichiometries of Pu­(IV) under the given conditions.

As was observed in the experiments with the An­(III) and An­(VI) pairs, a high gluconate concentration stabilizes the oxidation state + IV. Figure S12, Supporting Information shows the Pourbaix diagrams of Pu in the presence of different gluconate concentrations. The stability area of Pu­(IV) increases with increasing gluconate.

### 3.2 Comparison of Complexation Constants

The complexation of f-elements is of an electrostatic nature.^28^ Ligands with the same bonding motif often follow a linear trend in complexation strength based on the effective charge *z*_eff_ of the f-element. This is the case for the 1:1 acetate complexes of Pu­(III),^29^ Am­(III), Th­(IV),^26^ Np­(V), U­(VI),^30^ and Pu­(VI).^31^ Ca­(II)^32^ was added for comparison. In Figure 7, log β^0^ is plotted against the effective charge of each metal cation,^28^ exhibiting a linear trend. In general, EXAFS studies show a bidentate coordination of the acetate ligand to the actinide.^33–36^

**Figure 7:**
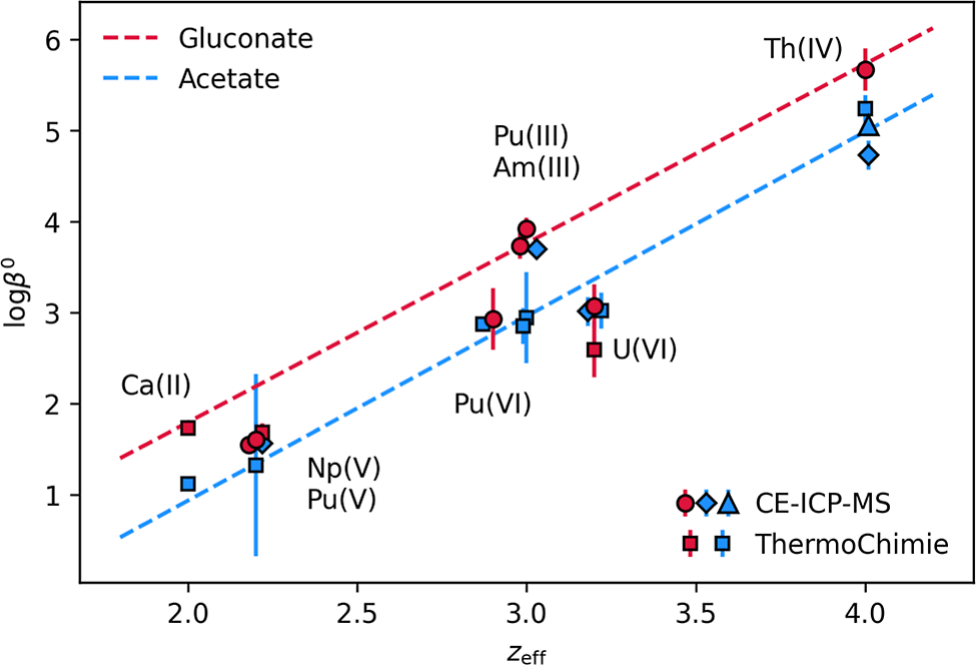
log β^0^ values of the 1:1 acetate and gluconate complexes plotted against the effective cationic charge.^28^ For better readability, overlapping points were shifted by *z*_eff_ = ±0.02. Values determined in this work are marked by circles, values determined using CE-ICP-MS by Willberger et al.^30^ and Lohmann et al.^26^ are marked by diamonds and triangles, respectively, and values taken from the ThermoChimie V13a database are marked by squares (references in Table S9, Supporting Information). Gluconate complexes are red and acetate complexes are blue.

For the gluconate complexation, an interesting effect is observed. Ca­(II), An­(III), and Th­(IV) show a linear trend with an increased complexation strength compared to acetate, while An­(V) and An­(IV) fall on the trend of acetate complexation. This was also noticed by Zhang et al. for U­(VI).^17^

An­(V) and An­(VI) are both present as actinyl moieties [AnO_2_]^z+^. Two covalently bonded oxygens are linearly arranged with the metal cation, which allows ligands to bind only in the equatorial plane. For An­(III) and An­(IV), gluconate is expected to form a stronger tridentate bond,^7,14^ which seems to be sterically hindered for An­(V) and An­(VI). The similar complexation constants for the acetate and gluconate complexes of An­(V) and An­(VI) suggest a similar bidentate bonding motif through the carboxylic function of both acid anions. This is supported by the EXFAS measurements of U­(VI)–GLU.^17^ It has to be noted that Willberger et al.^30^ determined a higher log β^0^ for [Am­(AcO)]^2+^ compared to previous literature, which in turn is similar to the log β^0^ for [Am­(GLU)]^2+^ determined in the present work.

## 4 Conclusions

Using the coupling between capillary electrophoresis and ICP-MS, it was possible to investigate the gluconate complexation of the major Pu oxidation states from (III) to (VI) as well as the redox stable actinides Am­(III), Th­(IV), Np­(V), and U­(VI). By addition of a redox stable actinide to the Pu sample, the oxidation state of Pu could be verified by comparing the electrophoretic mobilities. This way, the complex formation constants of three successive binary [An­(GLU)_*x*_]^3–*x*^ (*x* = 1–3) complexes could be determined for Am­(III) and Pu­(III). For Np­(V) and Pu­(V), the complex formation constants of the first binary [AnO_2_(GLU)]_(aq)_ complex were determined and those of the second [AnO_2_(GLU)_2_]^−^ complex were estimated. For U­(VI) and Pu­(VI), the constants of the [AnO_2_(GLU)]^+^, [AnO_2_(GLU_–H_)]_(aq)_, and [AnO_2_(GLU_–H_)­(GLU)]^−^ complexes were determined. Using CE-ICP-MS, it was possible to validate/confirm previous log β^0^ values for the complexation of the redox analogues Np­(V) and U­(VI), but at much lower actinide concentrations than before, i.e., at 2 × 10^–7^ M. The corresponding complexation constants for Pu were determined for the first time.

Plutonium in the oxidation states (III), (V), and (VI) behaved very similar to the corresponding redox analogous actinides. This was not the case for Th­(IV)/Pu­(IV). Here, the first five binary [Th­(GLU)_*x*_]^4–*x*^ (*x* = 1–5) complexes were determined for Th­(IV), whereas mixed Pu–OH–GLU complexes were proposed for Pu. The comparison of the first complex formation constants of the An–GLU complexes suggests a different bonding motif between An^3+/4+^ and AnO_2_^+/2+^, with AnO_2_^+/2+^ forming the weaker complexes.

## Supplementary Material



## Notes

Additional CE-ICP-MS data, speciation diagrams, experimental parameters, and electropherograms (PDF)
