# Supplementary material for: Complexation of Plutonium and Other Actinides in Different Oxidation States with Gluconate at Low pH Values—A CE-ICP-MS Study
Source: Inorg Chem. 2026 Feb 10;65(7):3806–14. doi: 10.1021/acs.inorgchem.5c04403 (PMC12933882; doi:10.1021/acs.inorgchem.5c04403)
Supplement: Supplementary file 1 [file ic5c04403_si_001.pdf]

SUPPORTING INFORMATION

Complexation of Plutonium and other Actinides in Different Oxidation States with Gluconate at low pH Values – a CE-ICP-MS Study

Janik Lohmann[1], Felix Sprunk[1], Diana Velikotrav[1], Alexander Wiebe[1], Julia Zemke[1], and Tobias Reich[1]*

[1]Johannes Gutenberg-Universität Mainz, Department of Chemistry - Nuclear Chemistry, 55099 Mainz, Germany.

The Supporting Information comprises 17 pages,

including 17 figures, 10 tables, and 13 reference.

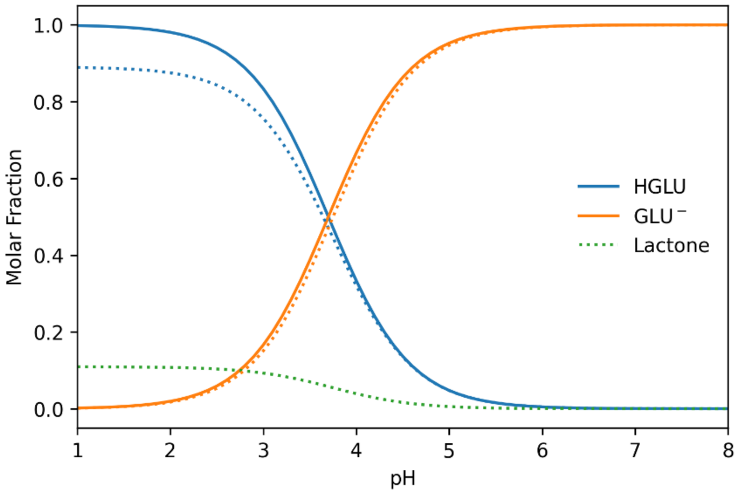

Figure S1. Speciation of gluconic acid at I = 0.1 M as function of pH calculated only using the pKa value (pK_a = 3.7) (solid lines) and under consideration of the lactonization (dotted lines)[1]. The free gluconate (GLU^-) fraction does not differ significantly between the two approaches.

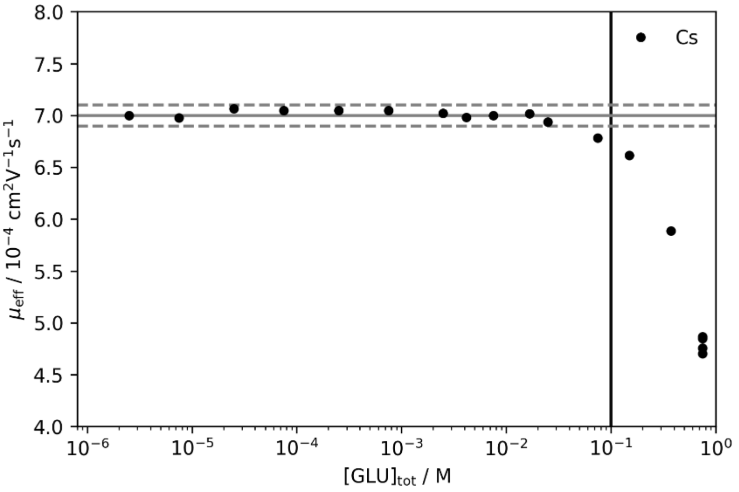

Figure S2. Measured electrophoretic mobility μ_{eff} of Cs^+ as a function of the total gluconate concentration $[GLU]_{tot}$. The data were collected as part of the investigation of the An(IV)-system in the pH range 1.30–2.76. No complexation with Cs^+ is expected in the free gluconate concentration range ($[GLU^-] < 0.1$ M) investigated. At $[GLU]_{tot} > 0.1$ M a significant decrease in electrophoretic mobility was observed coinciding with an increase in viscosity of the samples. The electrophoretic mobility is inversely proportional to the viscosity η of the solution ($\boldsymbol{\mu = \frac{q}{6\pi r\eta}}$). Therefore, a correction factor has been established to correct the measured mobilities for the change in viscosity: $\boldsymbol{f_\eta} = \frac{\overline{\mu_{Cs}}}{\mu_{Cs}}$. The average mobility of $\overline{\boldsymbol{\mu_{Cs}}} = \mathbf{7 \times 10^{-4}}$ cm^{2}V^{-1}s^{-1} was determined in the concentration range where the viscosity is only determined by 0.1 M $NaClO_4$. The $\boldsymbol{f_\eta}$ was applied to the mobilities of the other elements in the samples: $\boldsymbol{\mu_{korr} = \mu_{eff} \times f_\eta}$.

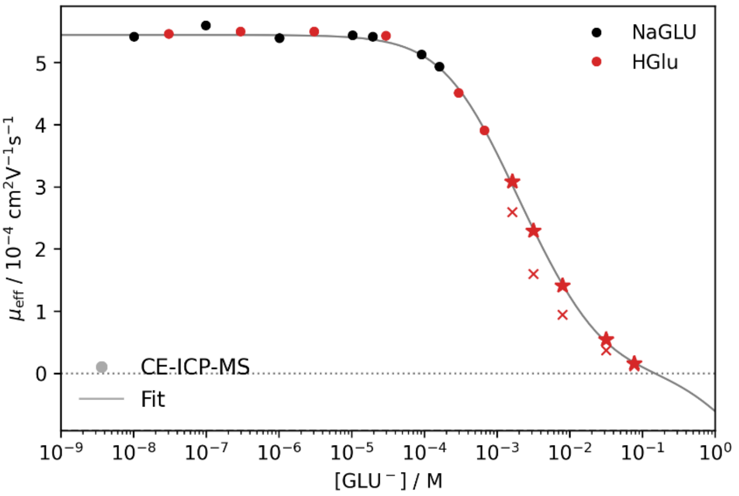

Figure S3. Measured electrophoretic mobility μ_{eff} of Eu(III) as a function of the free gluconate concentration $[GLU^-]$ in the pH range 1.30–2.76. At $[GLU]_{tot} > 0.1$ M ($[GLU^-] > 1 \times 10^{-3}$ M) mobilities (marked by x) were corrected (marked by stars) for the change in viscosity as described in Figure S2. Complex formation constants were determined as described in Zenker et al.[2] without considering lactonization. In addition, the data in this work and Zenker et al.[2] were retreated considering lactonization. The results are summarized in Table S1.

Table S1. Calculated complex formation constants log $\beta^{I=0.1\ M}$ for the Eu(III)-GLU system in this work and Zenker et al.[2]. Without considering lactonization, the experiment at pH 1.3−2.76 reproduced the complex formation constants determined at pH 4. Except for the $[Eu(GLU)_3]_{(aq)}$ complex, where the difference is more significant. This is most likely because of an incomplete compensation of the viscosity effect of the last two data points at 0.75 M HGLU. The influence of lactonization on the complexation constants lies close to or within the margin of error. Therefore, this proves lactonization is negligible.

	**No lactonization**		**Lactonization**	
**Species**	**log $\beta^{I=0.1\ M}$ pH 1.3 – 2.76**	**log $\beta^{I=0.1\ M}$ pH 4[2]**	**log $\beta^{I=0.1\ M}$ pH 1.3 – 2.76**	**log $\beta^{I=0.1\ M}$ pH 4[2]**
$[Eu(GLU)]^{2+}$	3.33 ± 0.04	3.33 ± 0.06	3.38 ± 0.04	3.35 ± 0.06
$[Eu(GLU)_2]^{+}$	6.02 ± 0.04	5.97 ± 0.05	6.12 ± 0.04	6.00 ± 0.05
$[Eu(GLU)_3]_{(aq)}$	7.96 ± 0.05	7.62 ± 0.07	8.11 ± 0.05	7.66 ± 0.07
$[Eu(GLU)_4]^{-}$	7.68 ± 0.48	7.63 ± 0.35	7.87 ± 0.48	7.67 ± 0.35

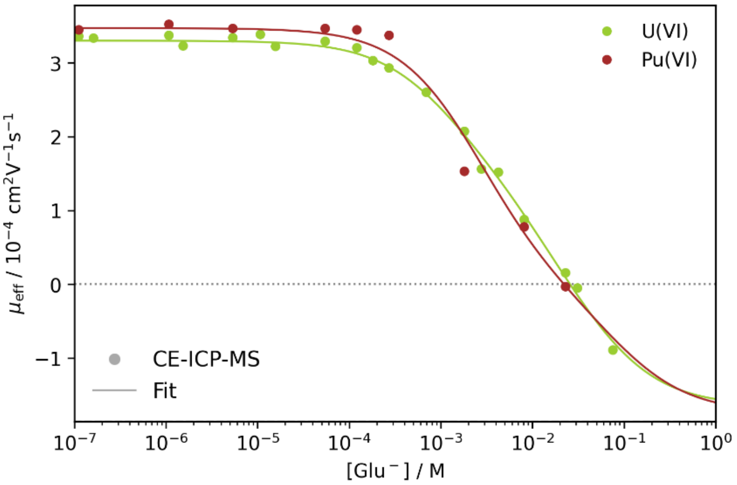

Figure S4. Plot of the measured electrophoretic mobilities μ_{eff} of ^{238}U(VI) and ^{239}Pu(VI) against the free gluconate concentration [GLU$^-$] at pH 3 and $I = 0.1$ M ($NaClO_4$). Fits include the 1:1 through 1:3 An(VI)–GLU complexes using eqs. 4-main and 5-main; $R^2_{U(VI)} = 0.997$ and $R^2_{Pu(VI)} = 0.980$.

Table S2. Calculated complex formation constants of the An(VI)-GLU complexes of U(VI) and Pu(VI) obtained from the fitting procedure (Figure S4) at $I = 0.1$ M ($NaClO_4$) and ϑ = 25°C. The lack of reliable data points for Pu(VI) due to the high tendency of reduction under the experiment parameters results in higher uncertainty for the formation constants.

**Reaction**	$\log \beta^{I=0.1\,\mathrm{M}}$	
	U(VI)	Pu(VI)
$\mathbf{AnO_2^{2+} + GLU^- \rightleftharpoons [AnO_2(GLU)]^+}$	2.98 ± 0.06	2.91 ± 0.25
$\mathbf{AnO_2^{2+} + 2GLU^- \rightleftharpoons [AnO_2(GLU)_2]_{(aq)}}$	4.96 ± 0.05	5.20 ± 0.14
$\mathbf{AnO_2^{2+} + 3GLU^- \rightleftharpoons [AnO_2(GLU)_3]^-}$	6.19 ± 0.07	6.27 ± 0.42

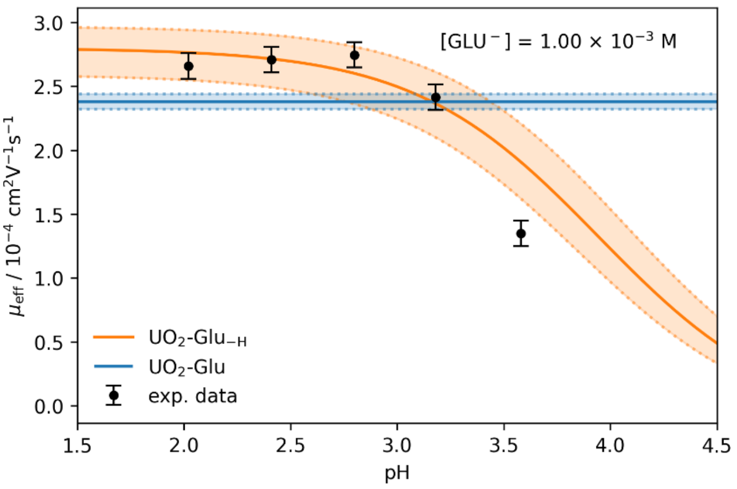

Figure S5. Plot of the measured electrophoretic mobility μ_{eff} of ^{238}U(VI) against pH at a constant free gluconate concentration [GLU⁻] of 1×10^{-3} M and $I = 0.1$ M ($NaClO_4$) (black). Measured electrophoretic mobilities are given in Table S8. The trend in mobility was predicted based on the formation of the binary UO_2-GLU complexes given in Table S2 (blue) or UO_2-GLU$_{-H}$ complexes listed in Table 3-main (orange). The shaded area gives the uncertainties. The trend in the measured mobility is better described by the formation of UO_2-GLU$_{-H}$ complexes. It is noted that both models intersect around pH 3, the pH value where the initial experiment was carried out.

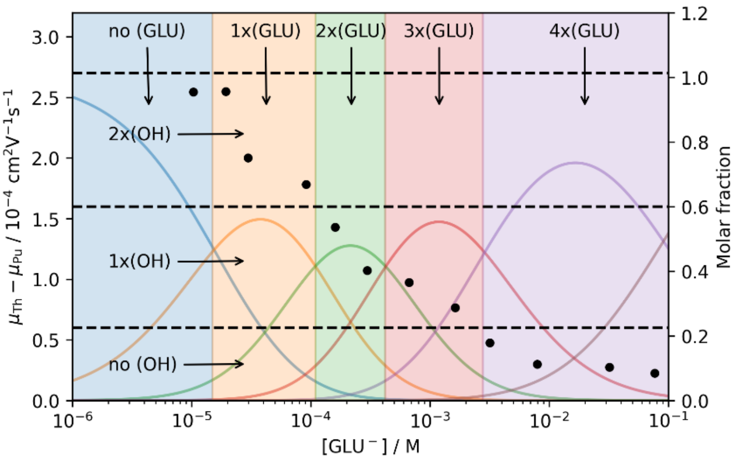

Figure S6. Difference in electrophoretic mobilities of Th(IV) and Pu(IV) as a function of free gluconate concentration [GLU$^-$] (black dots) as well as the speciation of Th(IV) calculated from the constants determined in this work (Table 4-main). For the possible Pu-OH-GLU complexes, the number of OH ligands was estimated based on the difference in mobility with 1.6×10^{-4} cm^{2}V^{-1}s^{-1} $\leq \Delta\mu < 2.7 \times 10^{-4}$ cm^{2}V^{-1}s^{-1} corresponding to two OH$^-$, 0.6×10^{-4} cm^{2}V^{-1}s^{-1} $\leq \Delta\mu < 1.6 \times 10^{-4}$ cm^{2}V^{-1}s^{-1} corresponding to one OH$^-$, and $\Delta\mu < 0.6 \times 10^{-4}$ cm^{2}V^{-1}s^{-1} corresponding to no OH ligands. The number of GLU ligands was estimated based on the speciation of Th(IV) as a similar chemical behavior is expected. This way, the five dominating Pu-OH-GLU complexes under the experimental conditions were determined to be: $[Pu(OH)_2]^{2+}$, $[Pu(OH)_2(GLU)]^{+}$, $[Pu(OH)(GLU)_2]^{+}$, $[Pu(OH)(GLU)_3]_{(aq)}$, $[Pu(GLU)_4]_{(aq)}$, and $[Pu(GLU)_5]^{-}$.

Table S3. Assumed electrophoretic mobilities μ_i of the individual species investigated. The mobilities were estimated based on the quotient $Q = \frac{z}{\mu}$ [10^4 Vs/cm^2] determined from the mobility μ [10^{-4} cm^{2}V^{-1}s^{-1}] and the ionic charge z of the free actinide ions $[An]^{z+}$. For simplicity, $[AnO_2]^{z+}$ is also written as $[An]^{z+}$.

	**Am(III)**	**Pu(III)**	**Th(IV)**	**Pu(IV)**	**Np(V)**	**Pu(V)**	**U(VI)**	**Pu(VI)**
**Q**	**0.68**	**0.67**	**0.77**	**0.77***	**0.46**	**0.45**	**0.61**	**0.58**
$[An]^{z+}$	4.42	4.47	5.18		2.18	2.23	3.30	3.47
$[An(GLU)]^{z-1}$	2.95	2.98	3.89		0.00	0.00	1.65	1.74
$[An(GLU)_2]^{z-2}$	1.47	1.49	2.59		−1.50	−1.50	0.00	0.00
$[An(GLU)_3]^{z-3}$	0.00	0.00	1.28				−1.65	−1.74
$[An(GLU)_4]^{z-4}$			0.00	0.00				
$[An(GLU)_5]^{z-5}$			−1.28	−1.28				
$[An(GLU_{-H})]^{z-2}$							0.00	0.00
$[An(GLU_{-H})(GLU)]^{z-3}$							−1.65	−1.74
$[An(OH)_2]^{z-2}$				2.59				
$[An(OH)_2(GLU)]^{z-3}$				1.28				
$[An(OH)(GLU)_2]^{z-3}$				1.28				
$[An(OH)(GLU)_3]^{z-4}$				0.00				

* For Pu(IV) the free Pu^{4+} was not observed, Q was therefore estimated based on Th(IV).

Table S4. Sample composition and electrophoretic mobilities determined for Am(III) and Pu(III).

pH	$[GLU]_{tot}$ / M	$[GLU^-]$ / M	μ_{eff} (Am(III)) / $cm^2V^{-1}s^{-1}$	μ_{eff} (Pu(III)) / $cm^2V^{-1}s^{-1}$	$\Delta\mu_{eff}$ / $cm^2V^{-1}s^{-1}$
3.98	9.0×10^{-7}	5.90×10^{-7}	4.44×10^{-4}	4.40×10^{-4}	0.07×10^{-4}
3.91	9.0×10^{-6}	5.57×10^{-6}	4.36×10^{-4}	4.66×10^{-4}	0.08×10^{-4}
3.61	2.7×10^{-5}	1.21×10^{-5}	4.58×10^{-4}	4.48×10^{-4}	0.08×10^{-4}
3.69	9.0×10^{-5}	4.45×10^{-5}	4.30×10^{-4}	4.36×10^{-4}	0.08×10^{-4}
3.87	2.7×10^{-5}	1.61×10^{-4}	4.20×10^{-4}	3.96×10^{-4}	0.08×10^{-4}
3.99	9.0×10^{-4}	5.95×10^{-4}	3.50×10^{-4}	3.59×10^{-4}	0.08×10^{-4}
4.16	2.7×10^{-3}	2.00×10^{-3}	2.82×10^{-4}	2.93×10^{-4}	0.07×10^{-4}
4.17	2.7×10^{-3}	2.02×10^{-3}	2.51×10^{-4}	-	0.08×10^{-4}
4.23	9.0×10^{-3}	6.95×10^{-3}	1.43×10^{-4}	1.49×10^{-4}	0.07×10^{-4}
4.28	9.0×10^{-3}	7.13×10^{-3}	1.21×10^{-4}	1.62×10^{-4}	0.06×10^{-4}
4.30	9.0×10^{-3}	7.19×10^{-3}	1.37×10^{-4}	-	0.08×10^{-4}
4.35	2.7×10^{-2}	2.21×10^{-2}	0.71×10^{-4}	0.74×10^{-4}	0.07×10^{-4}
4.34	9.0×10^{-2}	7.32×10^{-2}	0.25×10^{-4}	0.23×10^{-4}	0.06×10^{-4}

Table S5. Sample composition and electrophoretic mobilities determined for Eu(III), Th(IV), and Pu(IV). In blue: values corrected as described in figure caption S2.

pH	$[GLU]_{tot}$ / M	$[GLU^-]$ / M	μ_{eff} (Eu(III)) / $cm^2V^{-1}s^{-1}$	μ_{eff} (Th(IV)) / $cm^2V^{-1}s^{-1}$	μ_{eff} (Pu(IV)) / $cm^2V^{-1}s^{-1}$	$\Delta\mu_{eff}$ / $cm^2V^{-1}s^{-1}$
1.31	2.50×10^{-6}	1.01×10^{-8}	5.41×10^{-4}	4.38×10^{-4}	-	0.07×10^{-4}
1.31	7.50×10^{-6}	3.04×10^{-8}	5.46×10^{-4}	5.07×10^{-4}	-	0.08×10^{-4}
1.30	2.50×10^{-5}	9.91×10^{-8}	5.60×10^{-4}	5.26×10^{-4}	-	0.08×10^{-4}
1.30	7.50×10^{-5}	2.97×10^{-7}	5.50×10^{-4}	5.11×10^{-4}	-	0.07×10^{-4}
1.31	2.50×10^{-4}	1.01×10^{-6}	5.39×10^{-4}	5.11×10^{-4}	-	0.07×10^{-4}
1.31	7.50×10^{-4}	3.04×10^{-6}	5.50×10^{-4}	4.90×10^{-4}	-	0.07×10^{-4}
1.32	2.50×10^{-3}	1.04×10^{-5}	5.44×10^{-4}	4.70×10^{-4}	2.15×10^{-4}	0.07×10^{-4}
1.37	4.17×10^{-3}	1.94×10^{-5}	5.41×10^{-4}	4.40×10^{-4}	1.85×10^{-4}	0.07×10^{-4}
1.30	7.50×10^{-3}	2.97×10^{-5}	5.43×10^{-4}	3.75×10^{-4}	1.75×10^{-4}	0.08×10^{-4}
1.44	1.67×10^{-2}	9.13×10^{-5}	5.13×10^{-4}	3.45×10^{-4}	1.66×10^{-4}	0.07×10^{-4}
1.51	2.50×10^{-2}	1.60×10^{-4}	4.94×10^{-4}	2.81×10^{-4}	1.38×10^{-4}	0.06×10^{-4}
1.30	7.50×10^{-2}	2.97×10^{-4}	4.52×10^{-4}	2.20×10^{-4}	1.13×10^{-4}	0.07×10^{-4}
1.35	1.50×10^{-1}	6.67×10^{-4}	3.91×10^{-4}<br>3.70×10^{-4}	1.63×10^{-4}<br>1.72×10^{-4}	0.66×10^{-4}<br>0.70×10^{-4}	0.05×10^{-4}
1.34	3.75×10^{-1}	1.63×10^{-3}	3.08×10^{-4}<br>2.59×10^{-4}	0.98×10^{-4}<br>1.16×10^{-4}	0.21×10^{-4}<br>0.25×10^{-4}	0.05×10^{-4}
1.33	7.50×10^{-1}	3.19×10^{-3}	2.29×10^{-4}<br>1.59×10^{-4}	0.49×10^{-4}<br>0.70×10^{-4}	0.01×10^{-4}<br>0.02×10^{-4}	0.03×10^{-4}
1.73	7.50×10^{-1}	7.95×10^{-3}	1.41×10^{-4}<br>0.94×10^{-4}	0.14×10^{-4}<br>0.21×10^{-4}	-0.16×10^{-4}<br>-0.24×10^{-4}	0.03×10^{-4}
2.35	7.50×10^{-1}	3.21×10^{-2}	0.54×10^{-4}<br>0.37×10^{-4}	-0.21×10^{-4}<br>-0.31×10^{-4}	-0.49×10^{-4}<br>-0.72×10^{-4}	0.03×10^{-4}
2.76	7.50×10^{-1}	7.72×10^{-2}	0.16×10^{-4}<br>0.11×10^{-4}	-0.32×10^{-4}<br>-0.47×10^{-4}	-0.55×10^{-4}<br>-0.79×10^{-4}	0.03×10^{-4}

Table S6. Sample composition and electrophoretic mobilities determined for Np(V) and Pu(V).

pH	$[GLU]_{tot}$ / M	$[GLU^-]$ / M	μ_{eff} (Np(V)) / $cm^2V^{-1}s^{-1}$	μ_{eff} (Pu(V)) / $cm^2V^{-1}s^{-1}$	$\Delta\mu_{eff}$ / $cm^2V^{-1}s^{-1}$
**4.04**	9.0×10^{-7}	6.18×10^{-7}	2.16×10^{-4}	2.24×10^{-4}	0.07×10^{-4}
**3.93**	9.0×10^{-6}	5.66×10^{-6}	2.21×10^{-4}	2.26×10^{-4}	0.07×10^{-4}
**4.14**	9.0×10^{-5}	6.60×10^{-5}	2.21×10^{-4}	2.24×10^{-4}	0.07×10^{-4}
**4.24**	4.5×10^{-4}	3.49×10^{-4}	2.20×10^{-4}	2.26×10^{-4}	0.08×10^{-4}
**4.24**	9.0×10^{-4}	6.99×10^{-4}	2.20×10^{-4}	2.23×10^{-4}	0.08×10^{-4}
**4.28**	2.3×10^{-3}	1.78×10^{-3}	2.12×10^{-4}	2.15×10^{-4}	0.08×10^{-4}
**4.38**	4.5×10^{-3}	3.72×10^{-3}	2.05×10^{-4}	2.02×10^{-4}	0.08×10^{-4}
**4.34**	6.8×10^{-3}	5.49×10^{-3}	1.99×10^{-4}	2.05×10^{-4}	0.08×10^{-4}
**4.36**	9.0×10^{-3}	7.38×10^{-3}	1.92×10^{-4}	1.92×10^{-4}	0.08×10^{-4}
**4.30**	2.3×10^{-2}	1.80×10^{-2}	1.47×10^{-4}	1.44×10^{-4}	0.08×10^{-4}
**4.43**	4.5×10^{-2}	3.79×10^{-2}	1.04×10^{-4}	1.04×10^{-4}	0.07×10^{-4}
**4.39**	6.8×10^{-2}	5.61×10^{-2}	0.82×10^{-4}	0.80×10^{-4}	0.07×10^{-4}
**4.41**	9.0×10^{-2}	7.53×10^{-2}	0.60×10^{-4}	0.60×10^{-4}	0.07×10^{-4}

Table S7. Sample composition and electrophoretic mobilities determined for U(VI) and Pu(VI).

pH	$[GLU]_{tot}$ / M	$[GLU^-]$ / M	μ_{eff} (U(VI)) / $cm^2V^{-1}s^{-1}$	μ_{eff} (Pu(VI)) / $cm^2V^{-1}s^{-1}$	$\Delta\mu_{eff}$ / $cm^2V^{-1}s^{-1}$
2.85	9.0×10^{-7}	1.11×10^{-7}	3.36×10^{-4}	3.45×10^{-4}	0.06×10^{-4}
3.04	9.0×10^{-7}	1.62×10^{-7}	3.34×10^{-4}	*	0.06×10^{-4}
2.83	9.0×10^{-6}	1.07×10^{-6}	3.37×10^{-4}	3.53×10^{-4}	0.06×10^{-4}
3.01	9.0×10^{-6}	1.53×10^{-6}	3.23×10^{-4}	*	0.06×10^{-4}
2.83	4.5×10^{-5}	5.35×10^{-6}	3.35×10^{-4}	3.47×10^{-4}	0.06×10^{-4}
2.83	9.0×10^{-5}	1.07×10^{-5}	3.39×10^{-4}	-	0.06×10^{-4}
3.02	9.0×10^{-5}	1.56×10^{-5}	3.23×10^{-4}	*	0.06×10^{-4}
2.84	4.5×10^{-4}	5.46×10^{-5}	3.29×10^{-4}	3.47×10^{-4}	0.06×10^{-4}
2.84	1.8×10^{-4}	2.18×10^{-5}	3.09×10^{-4a}	2.99×10^{-4a}	0.06×10^{-4}
2.89	9.0×10^{-4}	1.21×10^{-4}	3.21×10^{-4}	3.45×10^{-4}	0.06×10^{-4}
3.10	9.0×10^{-4}	1.81×10^{-4}	3.03×10^{-4}	*	0.06×10^{-4}
2.95	1.8×10^{-3}	2.72×10^{-4}	2.94×10^{-4}	3.37×10^{-4}	0.06×10^{-4}
2.96	4.5×10^{-3}	6.93×10^{-4}	2.61×10^{-4}	-	0.06×10^{-4}
3.10	9.0×10^{-3}	1.81×10^{-3}	2.08×10^{-4}	1.53×10^{-4}	0.06×10^{-4}
3.35	9.0×10^{-3}	2.78×10^{-3}	1.56×10^{-4}	*	0.06×10^{-4}
3.19	1.8×10^{-2}	4.25×10^{-3}	1.52×10^{-4}	-	0.06×10^{-4}
3.04	4.5×10^{-2}	8.08×10^{-3}	0.88×10^{-4}	0.78×10^{-4}	0.06×10^{-4}
3.23	9.0×10^{-2}	2.28×10^{-2}	0.16×10^{-4}	-0.03×10^{-4}	0.06×10^{-4}
3.42	9.0×10^{-2}	3.10×10^{-2}	-0.05×10^{-4}	*	0.06×10^{-4}
4.41	9.0×10^{-2}	7.53×10^{-2}	-0.89×10^{-4}	*	0.06×10^{-4}

* No Pu(VI) was added to the samples. [a] Outlier

Table S8. Measured electrophoretic mobilities of U(VI) for the experiment in Figure S5 as well as pH values and the total and free gluconate concentrations $[GLU]_{tot}$ and $[GLU^{-}]$, respectively.

**pH**	**$[GLU]_{tot}$ / M**	**$[GLU^{-}]$ / M**	**μ_{eff} (U(VI)) / $cm^2V^{-1}s^{-1}$**
**2.02**	5.10×10^{-2}	1.04×10^{-3}	2.66×10^{-4}
**2.41**	2.10×10^{-2}	1.02×10^{-3}	2.71×10^{-4}
**2.80**	9.00×10^{-3}	1.01×10^{-3}	2.75×10^{-4}
**3.18**	4.20×10^{-3}	9.74×10^{-4}	2.42×10^{-4}
**3.58**	2.30×10^{-3}	9.92×10^{-4}	1.35×10^{-4}

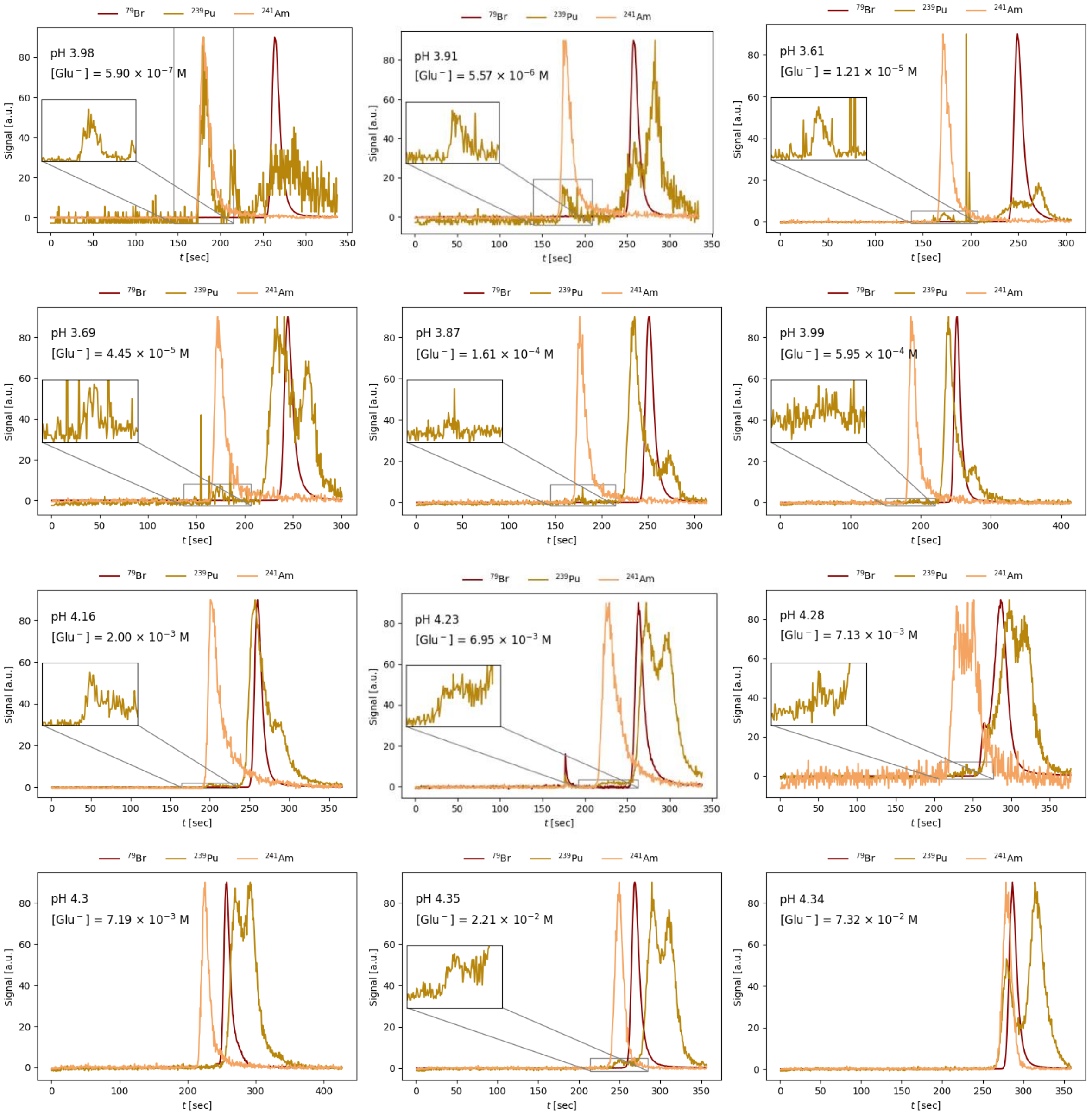

Figure S7. Electropherograms of $^{239}Pu(III)$, $^{241}Am(III)$, and ^{79}Br (EOF), $I = 0.1$ M ($NaClO_4$), varied [GLU], 25 °C, normalized signal, $l = 50$ cm, measured at 10 kV.

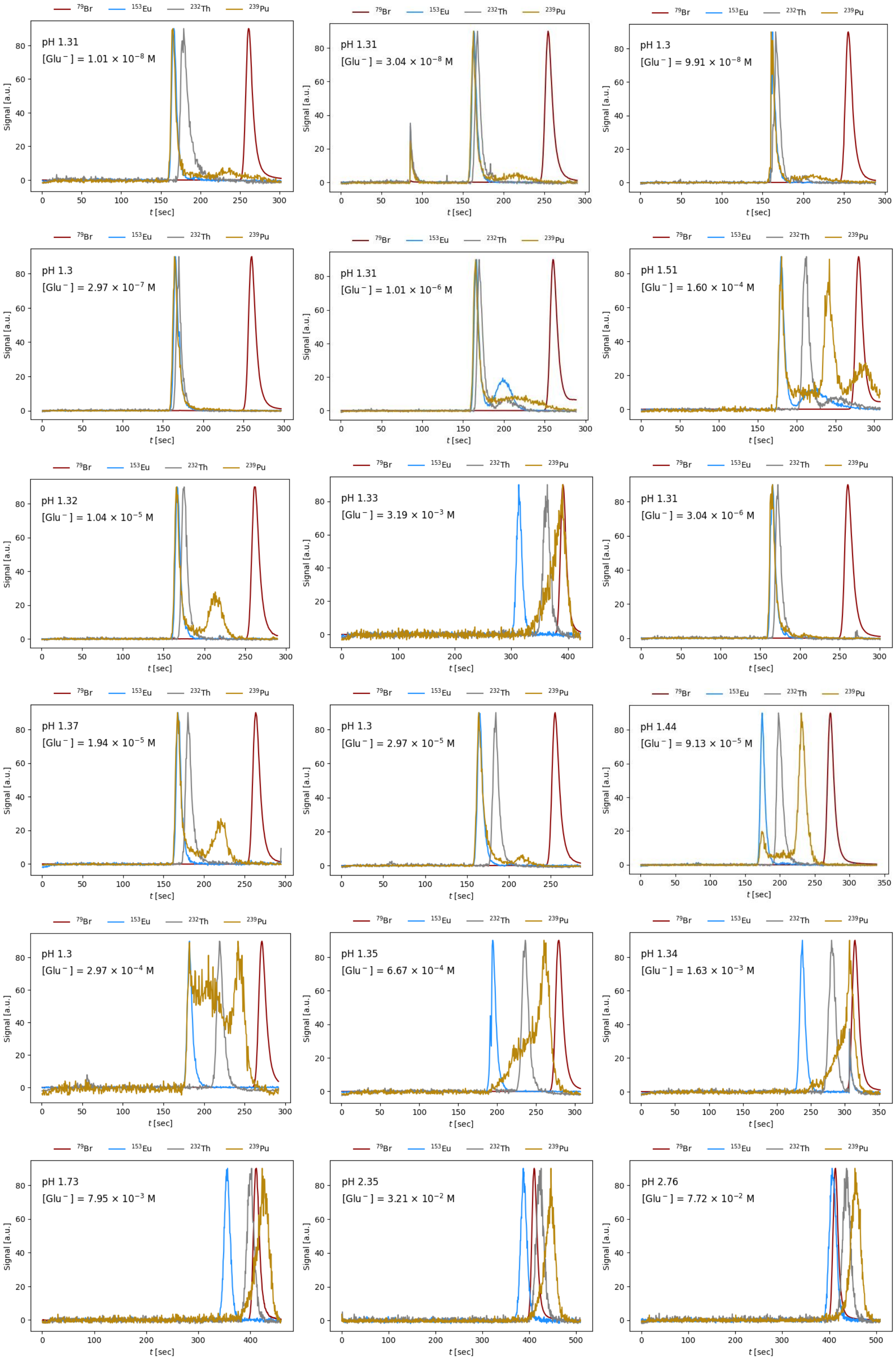

Figure S8. Electropherograms of ^{153}Eu(III), ^{232}Th(IV), ^{239}Pu(IV), and ^{79}Br (EOF), I = 0.1 M ($NaClO_4$), varied [GLU], 25 °C, normalized signal, l = 50 cm, measured at 10 kV.

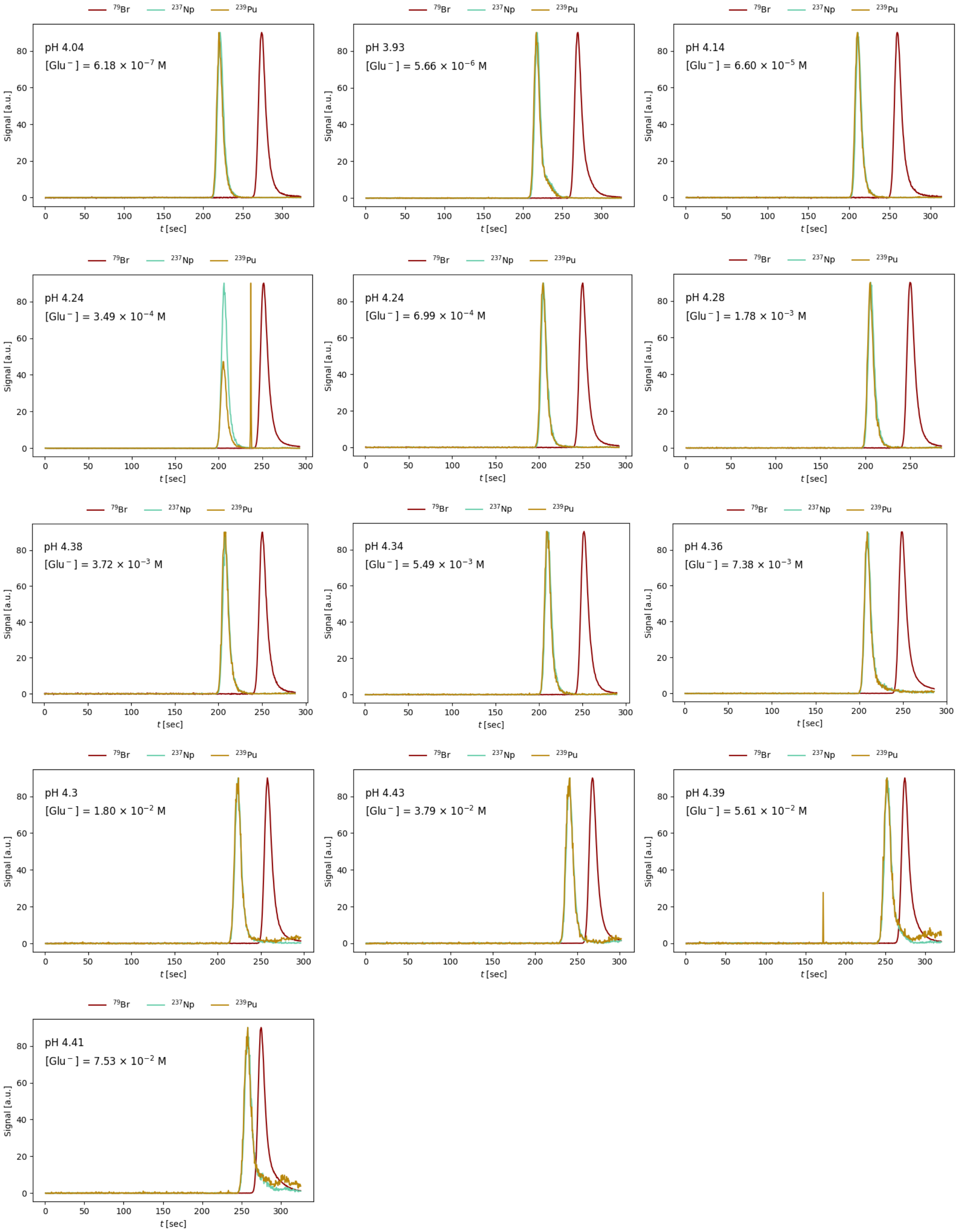

Figure S9. Electropherograms of ^{237}Np(V), ^{239}Pu(V), and ^{79}Br (EOF), I = 0.1 M ($NaClO_4$), varied [GLU], 25 °C, normalized signal, l = 50 cm, measured at 10 kV.

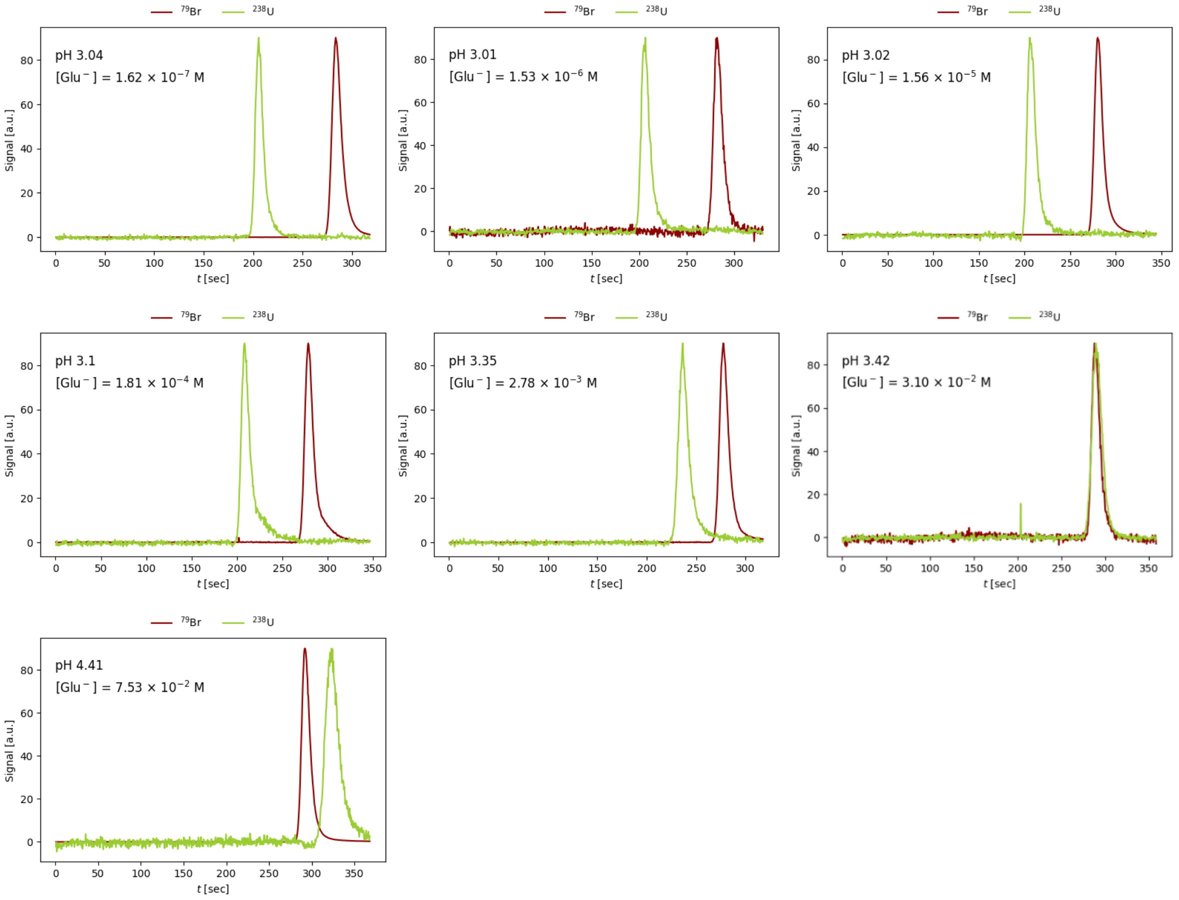

Figure S10. Electropherograms of ^{238}U(VI) and ^{79}Br (EOF), I = 0.1 M ($NaClO_4$), varied [GLU], 25 °C, normalized signal, l = 50 cm, measured at 10 kV.

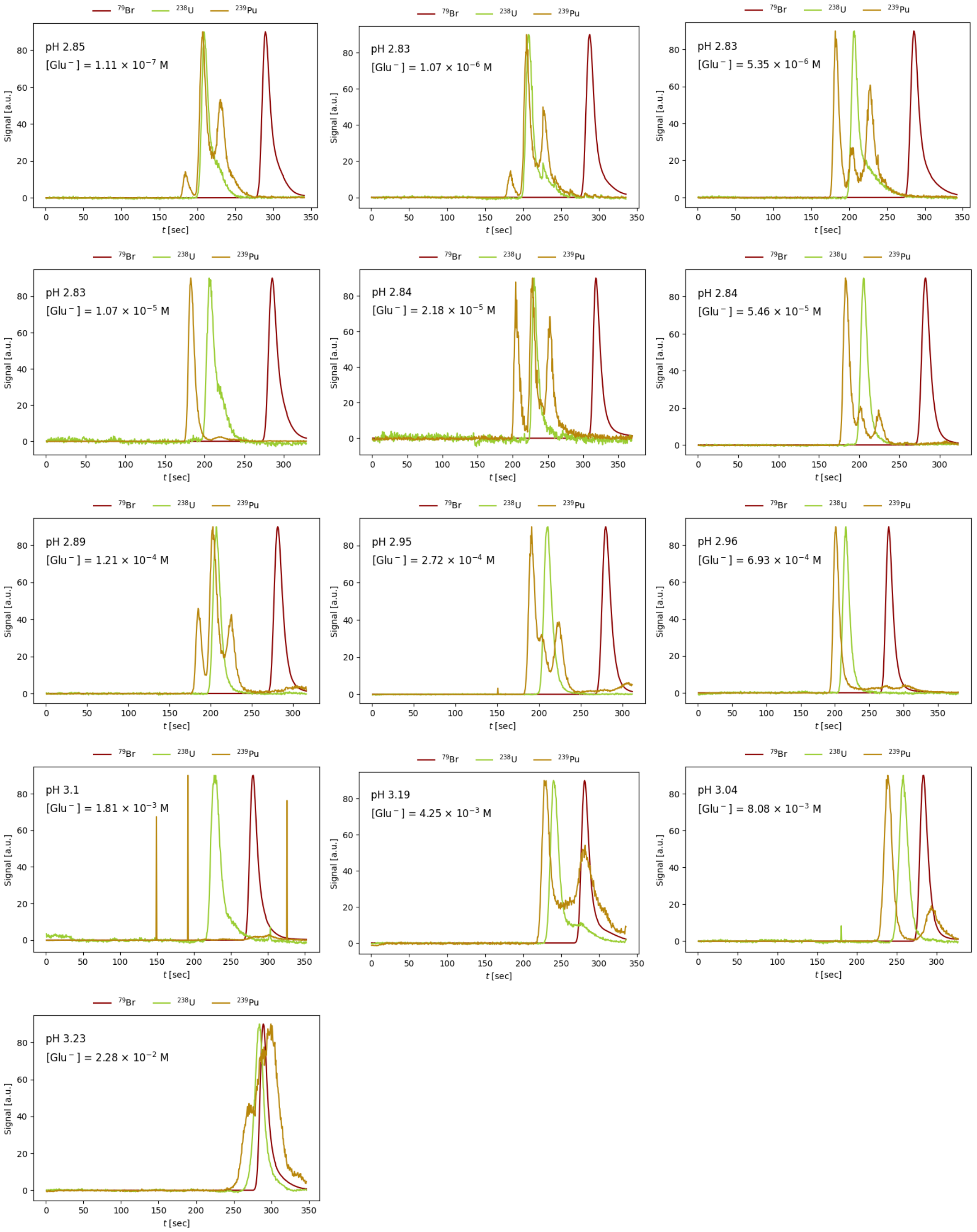

Figure S11. Electropherograms of ^{238}U(VI), ^{239}Pu(VI), and ^{79}Br (EOF), I = 0.1 M ($NaClO_4$), varied [GLU], 25 °C, normalized signal, l = 50 cm, measured at 10 kV.

Table S9. Complex formation constants $\log \beta^0$ of the gluconate (GLU^-) and acetate (AcO^-) complexes taken from literature.

Complex	$\log \beta^0$	Ref.
$[CaGLU]^+$	1.73 ± 0.05	3
$[NpO_2(GLU)]_{(aq)}$	1.68 ± 0.10	4
$[UO_2(GLU)]^+$	2.59 ± 0.30	5
$[CaAcO]^+$	1.12 ± 0.02	6
$[Pu(AcO)]^{2+}$	2.85 ± 0.20	7
$[Am(AcO)]^{2+}$	2.94 ± 0.50	8
$[Am(AcO)]^{2+}$	3.50 ± 0.09	9
$[Th(AcO)]^{3+}$	5.24 ± 0.15	8
$[Th(AcO)]^{3+}$	5.06 ± 0.11	10
$[Th(AcO)]^{3+}$	4.73 ± 0.16	9
$[NpO_2(AcO)]_{(aq)}$	1.32 ± 1.00	8
$[NpO_2(AcO)]_{(aq)}$	1.56 ± 0.03	9
$[UO_2(AcO)]^+$	3.02 ± 0.20	8
$[UO_2(AcO)]^+$	3.01 ± 0.12	9
$[PuO_2(AcO)]^+$	2.87 ± 0.02	8

Table S10. Ion interaction coefficients *ε(j,k)* [$kg \cdot mol^{-1}$] for cations/anions *j* with anions/cations *k* used for the extrapolation by the ThermoChimie database[11].

j	*k*	*ε(j,k)*
NpO_2^+	ClO_4^-	0.25
UO_2^{2+}	ClO_4^-	0.46
H^+	ClO_4^-	0.14
$UO_2(GLU)^+$	ClO_4^-	0
GLU^-	Na^+	−0.07
$NpO_2(GLU)_2^-$	Na^+	0

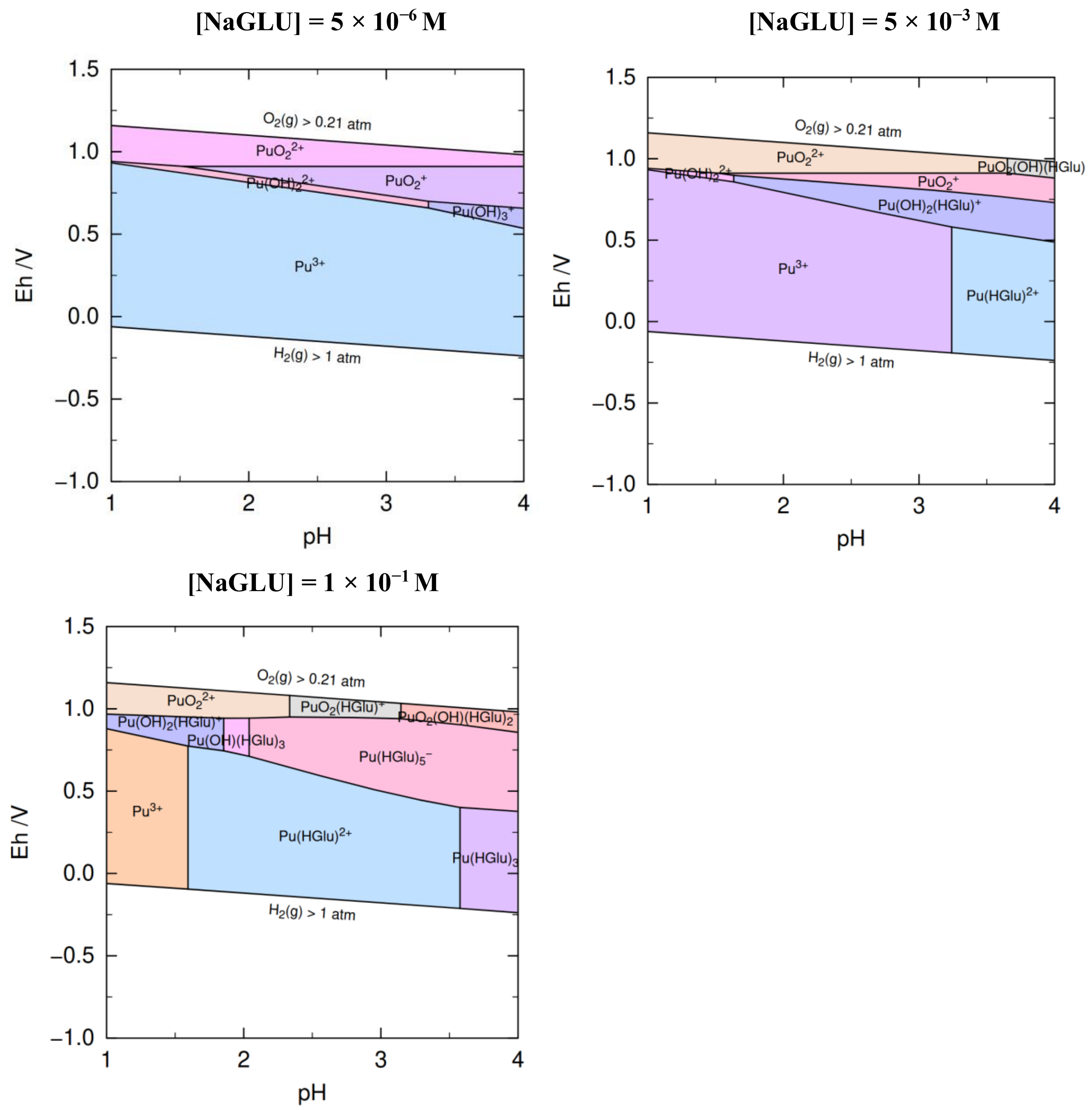

Figure S12. Pourbaix diagram of 1 × 10^{-7} M Pu at I = 0.1 M $NaClO_4$ and different gluconate concentrations, calculated using PhreePlot[12], Thermochimie version 13a[13] and the complex formation constants determined in the present work. The $Pu(OH)_3GLU$ complex was deactivated in the database as it did not match the experimental findings of the present work.

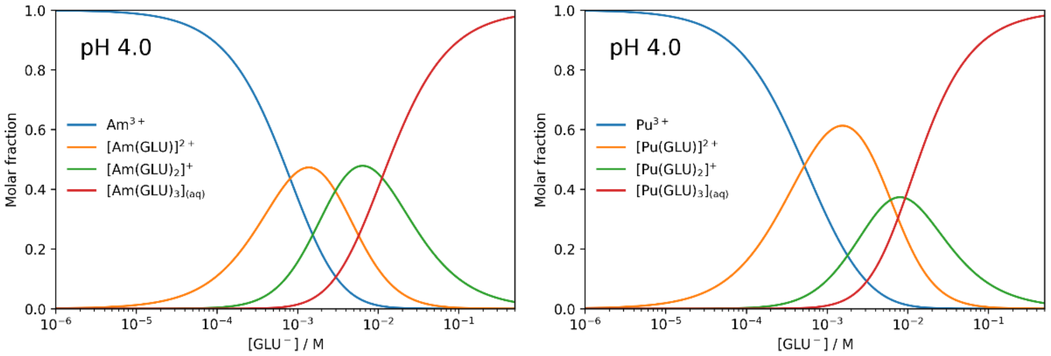

Figure S13. Speciation diagrams of An(III) against the free gluconate concentration [GLU$^-$] at I = 0.1 M. The species distribution was calculated manually based on the experimental parameters and complexation constants in the present work.

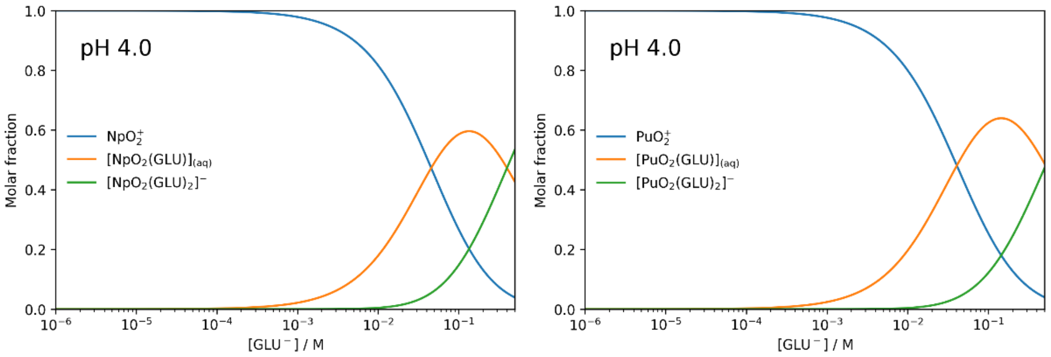

Figure S14. Speciation diagrams of An(V) against the free gluconate concentration [GLU$^-$] at I = 0.1 M. The species distribution was calculated manually based on the experimental parameters and complexation constants in the present work.

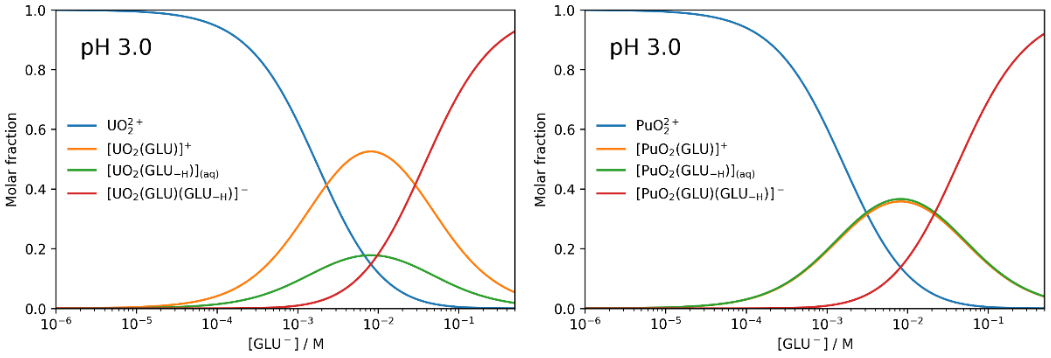

Figure S15. Speciation diagrams of An(VI) against the free gluconate concentration [GLU$^-$] at I = 0.1 M. The species distribution was calculated manually based on the experimental parameters and complexation constants in the present work.

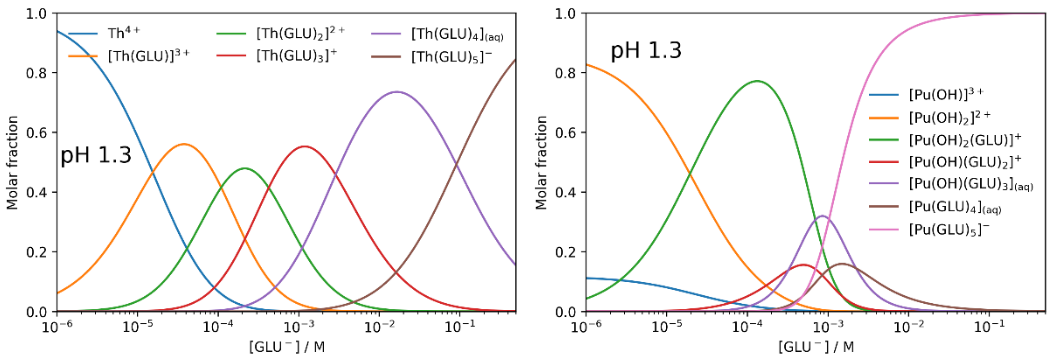

Figure S16. Speciation diagrams of An(IV) against the free gluconate concentration [GLU⁻] at I = 0.1 M. The species distribution was calculated manually based on the experimental parameters and complexation constants in the present work. The complexation constants of the Pu-OH complexes were taken from the ThermoChimie version 13a database[13] and extrapolated to an ionic strength of 0.1 M using the Davies equation.

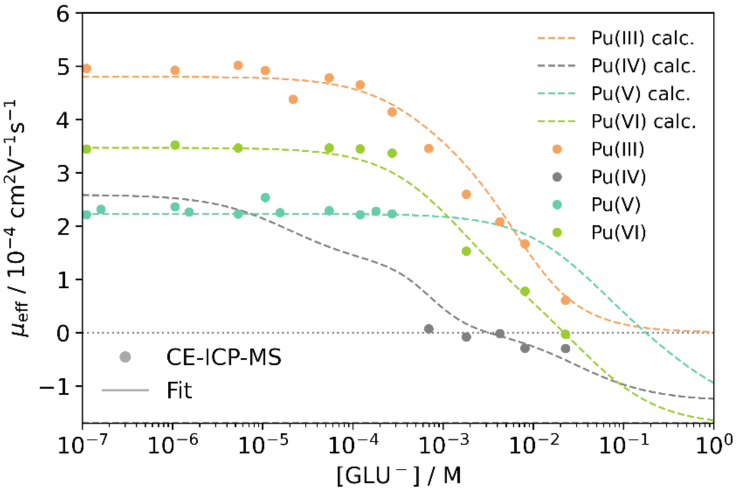

Figure S17. Electrophoretic mobilities of each Pu-peak from the An(VI) experiments (Fig. S11) as a function of free gluconate concentration [GLU⁻] compared to the trends in electrophoretic mobilities calculated in the main document (Figs. 3-main – 6-main). By comparing the measured electrophoretic mobilities to the mobilities observed in the other experiments, each peak can confidently be assigned to one of the four investigated oxidation states.

References

(1) Zubiaur, J.; Castaño, R.; Etxebarria, N.; Fernández, L. A.; Madariaga, J. M. Potentiometric Study of the Protonation and Distribution Equilibria of D-Gluconic-δ-Lactone Acid in Sodium Perchlorate Solutions at 25 °C and Construction of a Thermodynamic Model. *Talanta* **1998**, *45* (5), 1007–1014. DOI: 10.1016/s0039-9140(97)00207-5.

(2) Zenker, S.; Lohmann, J.; Chiorescu, I.; Krüger, S.; Kumke, M. U.; Reich, T.; Schmeide, K.; Kretzschmar, J. Complexation of Ln(III) Ions by Gluconate: Joint Investigation Applying TRLFS, CE-ICP-MS, NMR, and DF Calculations. *Inorg. Chem.* **2025**, *64* (16), 7970–7987. DOI: 10.1021/acs.inorgchem.4c05476.

(3) Schubert, J.; Lindenbaum, A. Stability of Alkaline Earth—Organic Acid Complexes Measured by Ion Exchange 1. *J. Am. Chem. Soc.* **1952**, *74* (14), 3529–3532. DOI: 10.1021/ja01134a021.

(4) Zhang, Z.; Clark, S. B.; Tian, G.; Zanonato, P. L.; Rao, L. Protonation of D-gluconate and its Complexation with Np(V) in Acidic to Nearly Neutral Solutions. *Radiochim. Acta.* **2006**, *94* (9-11), 531–536. DOI: 10.1524/ract.2006.94.9-11.531.

(5) Zhang, Z.; Helms, G.; Clark, S. B.; Tian, G.; Zanonato, P.; Rao, L. Complexation of Uranium(VI) by Gluconate in Acidic Solutions: a Thermodynamic Study with Structural Analysis. *Inorg. Chem.* **2009**, *48* (8), 3814–3824. DOI: 10.1021/ic8018925.

(6) Robertis, A. de; Di Giacomo, P.; Foti, C. Ion-Selective Electrode Measurements for the Determination of Formation Constants of Alkali and Alkaline Earth Metals with Low-Molecular-Weight Ligands. *Anal. Chim. Acta* **1995**, *300* (1-3), 45–51. DOI: 10.1016/0003-2670(94)00421-H.
(7) Moskvin, A. Complex Formation of Actinides with Anions of Acids in Aqueous Solutions. *Radiokhimiya* **1969** (11), 458–460.
(8) Richard , L., Grivé, M., Duro, L. Andra-TDB7 Task 3 – Organics. Selection of Formation Constants for Acetate Complexes of Ca and Radionuclides **2011**.
(9) Willberger, C.; Leichtfuß, D.; Amayri, S.; Reich, T. Determination of the Stability Constants of the Acetate Complexes of the Actinides Am(III), Th(IV), Np(V), and U(VI) Using Capillary Electrophoresis-Inductively Coupled Plasma Mass Spectrometry. *Inorg. Chem.* **2019**, *58* (8), 4851–4858. DOI: 10.1021/acs.inorgchem.8b03407.
(10) Lohmann, J.; Tamain, C.; Moisy, P.; Reich, T.; Aupiais, J. The Th-Acetate Chemical Equilibria: Is It Really That Simple? *Inorg. Chem.* **2025**, *64* (46), 22674–22682. DOI: 10.1021/acs.inorgchem.5c03418.
(11) J. Rodríguez-Mestres, E. Colàs, M. López-García and D. García. *TC Organics: Data Selection on Organic – RN Interactions*:*ThermoChimie Technical report - 3568_TC_Organics_A21_FR_vs2, 2023*.
(12) Parkhurst, D. L. User's guide to PHREEQC, a computer program for speciation, reaction-path, advective-transport, and inverse geochemical calculations **1995** (95-4227). DOI: 10.3133/wri954227.
(13) Madé, B.; Bower, W.; Brassinnes, S.; Colàs, E.; Duro, L.; Blanc, P.; Lassin, A.; Harvey, L.; Begg, J. D. Recent Developments in ThermoChimie – A Thermodynamic Database Used in Radioactive Waste Management. *Appl. Geochem.* **2025**, *180*, 106273. DOI: 10.1016/j.apgeochem.2024.106273.
